# Yeast Three-Hybrid Screen Identifies TgBRADIN/GRA24 as a Negative Regulator of *Toxoplasma gondii* Bradyzoite Differentiation

**DOI:** 10.1371/journal.pone.0120331

**Published:** 2015-03-19

**Authors:** Anahi V. Odell, Fanny Tran, Jenna E. Foderaro, Séverine Poupart, Ravi Pathak, Nicholas J. Westwood, Gary E. Ward

**Affiliations:** 1 Department of Microbiology and Molecular Genetics, University of Vermont, Burlington, Vermont, United States of America; 2 School of Chemistry and Biomedical Sciences Research Complex, University of St. Andrews and EaStCHEM, St Andrews, Fife, Scotland, United Kingdom; University at Buffalo, UNITED STATES

## Abstract

Differentiation of the protozoan parasite *Toxoplasma gondii* into its latent bradyzoite stage is a key event in the parasite’s life cycle. Compound 2 is an imidazopyridine that was previously shown to inhibit the parasite lytic cycle, in part through inhibition of parasite cGMP-dependent protein kinase. We show here that Compound 2 can also enhance parasite differentiation, and we use yeast three-hybrid analysis to identify TgBRADIN/GRA24 as a parasite protein that interacts directly or indirectly with the compound. Disruption of the Tg*BRADIN/GRA24* gene leads to enhanced differentiation of the parasite, and the Tg*BRADIN/GRA24* knockout parasites show decreased susceptibility to the differentiation-enhancing effects of Compound 2. This study represents the first use of yeast three-hybrid analysis to study small-molecule mechanism of action in any pathogenic microorganism, and it identifies a previously unrecognized inhibitor of differentiation in *T*. *gondii*. A better understanding of the proteins and mechanisms regulating *T*. *gondii* differentiation will enable new approaches to preventing the establishment of chronic infection in this important human pathogen.

## Introduction

Apicomplexan parasites, including those that cause malaria, toxoplasmosis and cryptosporidiosis, have developed a variety of strategies to persist in their hosts and achieve high transmission rates. These protozoan parasites have complex life cycles that typically include a sexual cycle in the definitive host and an asexual cycle in intermediate host(s). Some apicomplexans can form intracellular tissue cysts during their asexual cycle. The tissue cysts contain latent but highly infectious parasites surrounded by a cyst wall. *Toxoplasma gondii*, one of the most successful of the cyst-forming parasites, infects a wide range of intermediate hosts and can be horizontally transmitted between them by ingestion of tissue cysts without the need for passage through its definitive host [1]. The differentiation process that enables *T*. *gondii* to form tissue cysts is therefore of central importance to its life cycle.

During the initial acute phase of infection with *T*. *gondii*, tachyzoite-stage parasites invade and replicate rapidly, disseminating throughout the body. A strong adaptive immune response ultimately controls the acute infection but results in establishment of the chronic phase, wherein tachyzoites differentiate into bradyzoites and form tissue cysts, primarily in the brain and muscle. Tissue cysts can persist for the life of the host. However, if the individual becomes immunosuppressed, the infection can re-activate: bradyzoites differentiate back into tachyzoites, which replicate and disseminate, causing tissue lysis and inflammatory damage that can lead to blindness, encephalitis and death [2,3]. The mechanisms that regulate bradyzoite development are therefore of major clinical relevance.

Stress conditions and insults from the immune system are thought to be the natural inducers of tachyzoite-to-bradyzoite differentiation in infected hosts. Differentiation can also be experimentally induced in culture by heat shock [4], nutrient starvation [5], alkaline pH [6], and a wide variety of drugs including compounds that affect cyclic nucleotide signaling [7]. Analysis of the parasite transcriptome by microarray and SAGE (serial analysis of gene expression) has revealed large numbers of developmentally regulated genes [8–12]. Mutant parasite strains incapable of switching to the bradyzoite stage have been used to identify positive regulators of differentiation [10,12] and analyses of the epigenetic state of developmentally regulated genes [13–15] have shown that histone acetyltransferases, deacetylases and methyltransferases also affect expression of tachyzoite and bradyzoite-specific genes *in vitro*. These studies suggest that differentiation is a highly regulated process in which groups of genes are turned on and off in a specific temporal order [16]. Indeed, specific members of the ApiAP2 family of transcription factors are emerging as key regulators of the tachyzoite-to-bradyzoite developmental switch [17–19].

Compound 1 is a trisubstituted pyrrole initially identified in a cell-based screen designed to discover kinase inhibitors that block the growth of coccidian parasites *in vitro*. Compound 1 is a potent inhibitor of growth, attachment, invasion and motility of *T*. *gondii* (IC_50_ 0.23–1.2 μM; [20]), and was shown to directly inhibit parasite cGMP-dependent protein kinase (TgPKG) [21]. In addition, Compound 1 enhances tachyzoite-to-bradyzoite differentiation, likely through a host cell target [22].

**Fig 1:**
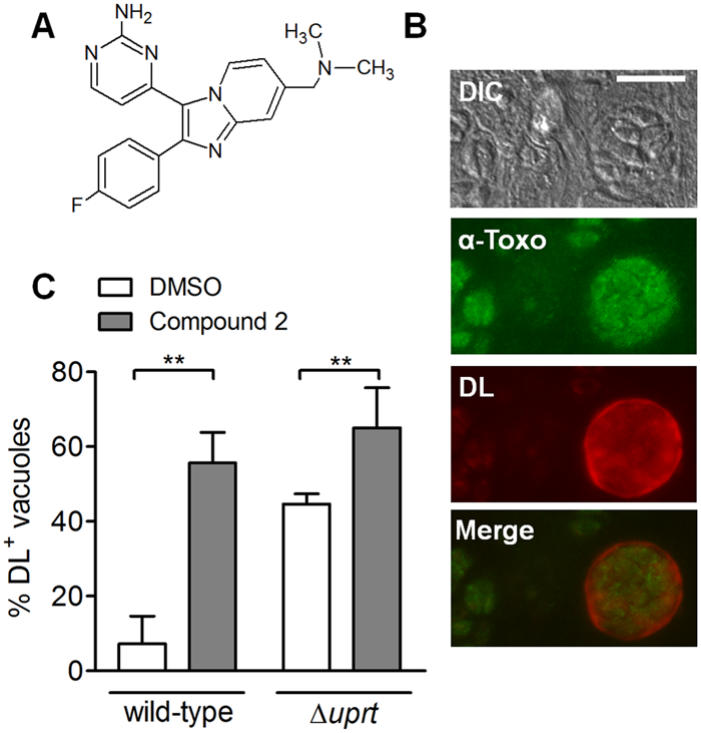
Compound 2 enhances differentiation. *A*. Structure of Compound 2. *B*. Immunofluorescence of a cyst-like structure using an anti-*Toxoplasma* antibody (green) to detect all parasites and *Dolichos biflorus* lectin (DL; red) to detect the cyst wall after 72 hr under CO_2_ starvation conditions. The corresponding differential interference contrast (DIC) and merged images are also shown. Scale bar = 10μm. *C*. Percentage of DL-positive vacuoles (DL+) in samples treated with either Compound 2 (3μM) or an equivalent volume of DMSO for 72 hr under CO_2_ starvation conditions (mean ± SD; n = 4 for the wild-type strain and n = 6 for Δ*uprt* strains). The data were compared using paired Student’s t-test (**p < 0.01).

In an effort to identify more potent inhibitors of coccidian PKG, a collection of structurally-related compounds was screened and a second small molecule, Compound 2 (Fig. 1A), was found to be an even more potent inhibitor of host cell invasion by *T*. *gondii* and related parasites (10–50nM; [23,24]). *In vitro*, Compound 2 was shown to be a potent inhibitor not only of TgPKG but also of Calcium Dependent Protein Kinase 1 (TgCDPK1) and Casein Kinase 1α (TgCK1α) [24]. The effect of Compound 2 on differentiation was not reported.

In the present study, we show that Compound 2 increases the rate of *T*. *gondii* tachyzoite-to-bradyzoite differentiation and enhances tissue cyst formation. In the first application of yeast three-hybrid analysis to small-molecule target identification in apicomplexan parasites, we identify TgBRADIN/GRA24 as a parasite protein that interacts with Compound 2. We show that TgBRADIN/GRA24 functions as an inhibitor of differentiation, and that Compound 2 exerts at least part of its differentiation-enhancing effect through a pathway that involves TgBRADIN/GRA24. These data enhance our understanding of the proteins involved in stage conversion in *T*. *gondii*, and may ultimately contribute to the development of new approaches to preventing tissue cyst formation and/or reactivation in human infections.

## Methods

### Differentiation assays

*T*. *gondii* parasites were cultured by passaging in human foreskin fibroblast (HFF; American Type Culture Collection [ATCC] CRL-1634) monolayers as described previously [25]. Confluent HFF monolayers in 6-well plates were infected with tachyzoites at a multiplicity of infection of 5x10^5^ and incubated in CO_2_ starvation media (Minimum Essential Medium lacking sodium bicarbonate but containing 1% v/v fetal bovine serum (FBS), 25mM HEPES, 20U/ml Penicillin, 20μg/ml Streptomycin and 2mM L-Alanyl-L-Glutamine dipeptide) at 37°C with 0.03% CO_2_ for 72 hr. The coverslips were fixed in phosphate buffered saline (PBS) containing 4% v/v paraformaldehyde for 20 min, permeabilized in PBS containing 0.25% v/v Triton X-100 for 15 min and blocked in PBS containing 1% w/v bovine serum albumin for 1 hr. Coverslips were incubated for 30 min with rabbit polyclonal anti-*Toxoplasma* (catalog #90700556; AbD Serotec, Raleigh NC), diluted 1:2000 in blocking solution. Samples were then incubated with goat anti-rabbit IgG conjugated to Alexa 488 (Invitrogen, Grand Island NY) at a 1:500 dilution together with TRITC-conjugated *Dolichos biflorus* lectin (Sigma-Aldrich, St. Louis MO) at a dilution of 1:100 for 60 min. Alternatively, samples were incubated with rabbit anti-HSP30/BAG1 antibody (generously provided by Dr. Sergio Angel) at a dilution of 1:100 and mouse anti-IMC1 (MAb 45.36 at 0.75 μg/ml) followed by goat anti-rabbit IgG conjugated to Alexa 546 (Invitrogen) and goat anti-mouse IgG conjugated to Alexa 488, each at 1:500. The coverslips were imaged at 100X on a Nikon Eclipse TE300 epifluorescence microscope; 300–600 vacuoles were counted blind along the transverse axis of each coverslip.

Differentiation assays in which the parasites were treated with Compound 2 post-infection were done as described above with the modification that parasites were allowed to invade the monolayer in Dulbecco’s Minimum Essential Medium (supplemented with 1% v/v FBS, 5mM HEPES, 20U/ml Penicillin and 20μg/ml Streptomycin) for 1 hr at 37°C with 5% CO_2_. The infected monolayers were washed three times with fresh medium and the medium was replaced with CO_2_ starvation medium containing either Compound 2 or the equivalent amount (0.25% v/v) of DMSO. Plates were incubated for 72 hr at 37°C with 0.03% CO_2_ and processed for fluorescence microscopy as described above.

We observed that the percentage of vacuoles in an infected HFF monolayer that form cyst-like structures under CO_2_ starvation conditions is dependent upon host cell passage number, with higher levels of differentiation in older cells (data not shown). Similarly, treatment of infected monolayers with 0.25% v/v DMSO (vehicle) under CO_2_ starvation conditions also enhances differentiation, explaining the differences seen in the induction level of wild-type parasites with and without DMSO (compare Figs. 1C and 6A).

### Synthesis of Compound 2 and MTX-Cmpd2.1

Compound 2 was synthesized using a modified version of a previously published procedure ([26]; see Scheme I in S1 Methods for details). The synthesis of MTX-Cmpd2.1 was achieved in 3 main steps: (1) synthesis of the Compound 2–linker fragment (Scheme II in S1 Methods); (2) synthesis of ^*t*^butyl-methotrexate (^*t*^Bu-MTX) (Scheme III in S1 Methods) and (3) coupling of these 2 fragments followed by ^*t*^butyl ester hydrolysis (Scheme IV in S1 Methods). An analogous approach to MTX-Cmpd1.1 was also taken (Scheme V in S1 Methods).

### Cloning of Compound 2 targets in the Y3H vector

The coding sequence for Tg*CK1α* was PCR amplified from our oligo(dT)-primed cDNA library using primers P1 and P2 (S1 Table). The PCR product (1kb) was then subcloned into pGEM-T Easy (Promega, Madison WI) following manufacturer’s instructions. The Tg*CK1α* insert was excised from the subcloning vector using MfeI and ligated into the EcoRI site of the Y2H vector pJG4–5 [27]. This places the insert in frame with the B42 activation domain (AD) encoded in pJG4–5. The orientation of the insert was verified by restriction digest.

The Tg*PKG* coding sequence corresponding to isoform 2 (M102 to the stop) [28] was amplified from cDNA using primers P3 and P4 (S1 Table). The cDNA was prepared from RH tachyzoite total RNA using Accuscript reverse transcriptase (Agilent Technologies, Santa Clara CA) following manufacturer’s instructions. The PCR product was subcloned into pGEM-T Easy and then cloned in pMW102 [29] at the EcoRI site.

The coding sequence for Tg*CDPK1* was amplified from cDNA using primers P5 and P6 (S1 Table). The sequence added to the 5’ end of the primers shares homology to sequence flanking the EcoRI site in the linearized plasmid pJG4–5, and once in the yeast cell these two DNA molecules will recombine to regenerate a circular plasmid [30]. The 1.5kb PCR fragment was co-transformed into yeast strain V784Y along with EcoRI-linearized and dephosphorylated pJG4–5. Yeast transformation was performed using the LiAc/ssDNA/PEG method [31]. Plasmid was isolated from yeast grown on synthetic complete media with glucose and lacking tryptophan (SC +Glu-trp) to select for clones containing the recombinant plasmid. Positive clones were confirmed by PCR. Plasmid was isolated using an adaptation of the bacterial miniprep protocol (Promega): cells were lysed by two freeze-thaw cycles, followed by incubation in 150mM NaCl, 1% w/v SDS and 0.05% v/v Tween 20 and phenol-chloroform extraction prior to neutralization of the yeast extract.

The C-terminal portion of isoform Tg*BRADIN-a* was amplified using primers P7 and P8 (S1 Table). The 676bp fragment was subcloned into pGEM-T Easy, digested with EcoRI and ligated at the EcoRI site in pJG4–5. Orientation of the insert in the final construct was confirmed by restriction digest.

### Reporter activation assays

Growth assays to test for the activation of the *LEU2* reporter in yeast strain V784Y were done both in plate and liquid culture. In-plate assays were done by spotting 1μl of yeast suspension onto dropout plates (SC +Gal/Raff-his-trp-ura-leu) containing 2% w/v galactose and 1% w/v raffinose in order to induce the Gal1 promoter that drives the expression of the prey/target fusion protein. The plates were spread with the appropriate amount of CID dissolved in DMSO using sterile glass beads the day prior to spotting the yeast, and kept at 21°C until ready to use. The yeast suspension was prepared by resuspending a fixed amount of yeast with a sterile pipet tip in 100μl of sterile double-distilled water. Plates were incubated at 30°C for 2–6 days wrapped with parafilm. Liquid growth assays were performed by diluting the yeast from a saturated overnight culture to 2x10^5^ cells/well in a sterile flat-bottom clear 96-well plate (Becton-Dickinson, Franklin Lakes NJ) with 150μl of the appropriate selective media containing either CID or the equivalent amount of DMSO. The plates were wrapped with parafilm and incubated at 30°C with shaking at 80rpm. Every 24 hr the absorbance at 600nm was measured using a pre-heated plate reader (BioTek, Winooski VT). Cell suspensions were mixed by pipetting prior to each measurement.

To measure activation of the *LacZ* gene, the standard liquid β-Galactosidase assay as described in the Yeast Protocols Handbook (PT3024–1; Clontech, Mountain View CA) was adapted to 150μl cultures grown in 96-well plates. Briefly, saturated overnight cultures were diluted 1:100 in SC +Gal/Raff-his-trp-ura media in sterile flat-bottom 96-well plates and incubated at 30°C for 72 hr. Yeast cultures were collected and centrifuged at 21°C at 16000xg for 5 min. The pellets were washed once with double-distilled water and resuspended in freshly prepared breaking buffer containing 1x protease inhibitor mix (P8340; Sigma-Aldrich, St. Louis MO), 100mM Tris-HCl pH8.0, 1mM DTT and 20% v/v glycerol. Acid-washed beads were added to each tube to fill the volume up to the meniscus and the cells were broken by 10 x 15 sec pulses of vortexing, each pulse followed by 1 min incubation on ice. The spheroblast suspension was then solubilized in 0.1% w/v SDS for 10 min at 21°C. 25μl of each lysate was used to measure total protein concentration by the standard microtiter Bradford assay (BioRad, Hercules CA). The rest of the extract was used for the β-galactosidase assay using CPRG (Roche, Indianapolis IN) as substrate, as described in the Yeast Protocols Handbook (Clontech).

### cDNA library generation

The *Toxoplasma* cDNA library was generated using mRNA from RH strain extracellular tachyzoites. Total RNA (762μg) was extracted from 100 T75 flasks of freshly egressed parasites using the animal cell protocol of the RNeasy kit (QIAGEN, Valencia CA). The quality of the extracted total RNA was assessed by chromatography with the 2100 Agilent Bioanalyzer (Agilent Technologies, Santa Clara CA). RNA preparations containing more than 5% contamination of host cell-derived RNA were discarded. The mRNA was isolated, retrotranscribed to cDNA and ligated into the library vector pJG4–5 at the EcoRI and XhoI sites by Express Genomics (Baltimore, MD). This cDNA library contained 3.51 x 10^7^ total cfu with an average insert size of 1.4kb.

### Yeast three-hybrid screens

The cDNA library was used to transform competent V784Y yeast using a library scale transformation as previously described (Clontech Matchmaker GAL4 Two-Hybrid System 3 & Libraries User Manual and ref. [32]). The transformation mix was then plated on 50 x 15mm dropout plates (SC +Glu-his-trp-ura). Transformants were collected, pooled, aliquotted and stored at -80°C. The plates were spread with CID using sterile glass beads the day before the start of the screen to give the final desired concentration. For each Y3H screen, one aliquot of yeast transformed with the cDNA library (~1 x 10^10^ cells) was thawed and diluted 1:10 in SC +Gal/Raff-his-trp-ura media and incubated with shaking at 30°C for 4 hr to induce the Gal1 promoter. The cell suspension was diluted in SC +Gal/Raff-his-trp-ura-leu to give an OD_600_ of approximately 0.3 and plated using sterile glass beads onto dropout plates (SC +Gal/Raff-his-trp-ura-leu +CID). As a negative control, library transformed yeast were also plated on one equivalent dropout plate spread with DMSO. Plates were inverted and incubated 3–5 days at 30°C. The screen was terminated when growth appeared on the negative control plate.

### Yeast Colony Hybridization

Radioactive probes were synthesized by PCR as previously described [33], using Thermopol Buffer, Taq Polymerase (New England Biolabs, Ipswich MA) and 20μM each of dATP/dTTP/dGTP supplemented with 3.3μM α^32^P-dCTP (3000Ci/mmol; MP Biomedicals, Santa Ana CA). The primers used to amplify/label the probes were P9 and P10 for the Tg*CDPK1* probe and P11 and P12 for the Tg*BRADIN* probe (S1 Table). The Tg*CDPK1* labeled probe (1.2kb) covered the 5’ end of the Tg*CDPK1* coding sequence. The Tg*BRADIN* labeled probe (0.8kb) covered the 3’ of the Tg*BRADIN-b* coding sequence. The labeled probes were purified using a PCR purification kit (QIAGEN) to remove unincorporated dNTPs. The labeling efficiency was 2x10^7^ cpm/μg DNA.

Yeast colonies from each hit in the Y3H screens were grown on dropout plates with the appropriate selection at 30°C for 1–2 days and subjected to colony hybridization with the Tg*CDPK1* and Tg*BRADIN* probes. The protocol used for hybridization was adapted from a previously described method [34]. Yeast colonies were lifted on Hybond N+ nylon membrane (Amersham Biosciences, Pittsburgh PA) and the membrane incubated on filter paper soaked with spheroblasting solution (1M sorbitol, 20mM EDTA, 10mM Tris-HCl, 14mM beta-mercaptoethanol and 100U/ml Zymolyase 20T (Seikagaku Corporation, Tokyo, Japan)) overnight at 37°C. The degree of cell wall breakage was assessed by visualization under the microscope. The membrane was then incubated for 10 min in each of the following solutions (colony side up on soaked sheets of filter paper): denaturing solution (1.5M NaCl, 0.5M NaOH); neutralizing solution (1.5M NaCl, 0.5M Tris-HCl pH 7.4); 0.5M Tris-HCl/6x sodium chloride/sodium citrate solution (SSC); and 2xSSC. The membrane was air-dried between incubations. UV-crosslinking of the DNA to the membrane was performed using a Stratalinker 1800 (Agilent Technologies).

The membrane was pre-hybridized for at least 1 hr at 65°C in hybridization solution (1% w/v BSA, 1mM EDTA, 0.5M NaHPO_4_ pH 7.2, 7% w/v SDS). The radioactive probe was boiled and diluted to 0.5–1x10^6^ cpm/ml in hybridization solution with 2mg of sonicated salmon sperm DNA. The hybridization of the membrane with the probe was done in a sealed bag at 65°C overnight. The membrane was washed three times with 250ml of low stringency wash buffer (0.5% w/v BSA, 1mM EDTA, 40mM NaHPO_4_ pH 7.2, 5% w/v SDS) at 23°C and three times with high stringency wash buffer (1mM EDTA, 40mM NaHPO_4_ pH 7.2, 1% w/v SDS) at 65°C. Membranes were exposed on BioMax MR film (Kodak, Rochester NY) at -80°C for 24–48 hr.

### Tg*BRADIN* targeting construct

Flanking sequences to generate the Tg*BRADIN* targeting construct were amplified using primers P13 and P14 for the 5’ flanking region (608bp) and P15 and P16 for the 3’ flanking region (687bp) (S1 Table). Flanks were amplified from RH tachyzoite genomic DNA (gDNA) extracted using DNAzol Reagent (Invitrogen) followed by ethanol precipitation. The PCR products were digested: with HindIII and KpnI (5’ flank) or BamHI and XbaI (3’ flank). Each fragment was then ligated into the respective sites in pGRA1ble [35]. The resulting plasmid (100μg) was digested with KpnI/XbaI and used for transfection of wild-type parasites (RH Δ*ku80* Δ*hxgprt*; see S2 Table)

### Tg*BRADIN* isoform PCR amplification

PCR of tachyzoite cDNA to amplify the 3’ end of Tg*BRADIN* isoforms was performed using primers P7 and P8 for Tg*BRADIN-a* and P17 and P18 for Tg*BRADIN-b* (S1 Table).

### *T*. *gondii* transfection and selection

Transfections were performed by electroporating 2 x 10^7^ wild-type parasites (model BTX ECM630 electroporator) resuspended in Cytomix Buffer (120mM KCl, 0.15mM CaCl_2_, 10mM potassium phosphate pH 7.6, 25mM HEPES-KOH pH 7.6, 2mM EDTA, 5mM MgCl_2_) supplemented with 2mM ATP and 5mM reduced glutathione. After transfection, enrichment of the Δ*bradin* parasites was done by two rounds of selection in the presence of 50μg/ml and 5μg/ml phleomycin as described previously [35]. Individual clones were isolated in 96-well plates by diluting the population and seeding each well with an estimated three parasites per well. Homologous recombination events were evaluated by PCR on gDNA, extracted from parasites using DNAzol reagent followed by ethanol precipitation. The PCRs used to evaluate the knockout are shown in S1 Fig (primers P19 and P20 for PCR A, and P19 and P21 for PCR B, S1 Table).

Wild-type and Δ*bradin* parasites were transfected with pUPRTKO plasmid digested with BglII as previously reported [36] to generate the corresponding Δ*uprt* and Δ*bradin* Δ*uprt* parasite lines (S2 Table). Parasites resistant to 5μM 5’-fluorodeoyuridine (FUDR) were obtained after two rounds of selection and clones isolated as described above. Recombination was confirmed with gDNA extracted from individual clones using a previously described PCR approach (primers P22 and P23 [36] for PCR C, S1 Table).

### Southern blot analysis of Δ*bradin* parasites

Genomic DNA was extracted from wild-type and Δ*bradin* parasites as above. 7.5ug of DNA was digested with SspI and RNAseA overnight. The digested DNA was electrophoresed in a 0.7% w/v agarose gel and transferred onto Hybond N+ nylon membrane by capillary transfer in alkaline solution [37].

Biotinylated probes were synthesized by PCR using Thermopol Buffer with Taq polymerase (New England Biolabs), 20μM each of dATP/dCTP/dGTP supplemented with a 50/50% mix of dTTP/biotinylated-dUTP (20μM final concentration) and gDNA (for probe 1) or plasmid pGRA1ble (for probe 2) as template. The primers used to amplify/label probe 1 were P19 and P20, which generate a 0.9kb fragment, and P24 and P25 for probe 2, which amplify the ble^R^ coding sequence yielding a 0.35kb product (S1 Table). The labeled probes were purified using a PCR purification kit (QIAGEN).

The membrane was pre-hybridized for at least 1 hr at 64°C in hybridization solution (see above). Biotinylated probe 2 was boiled and diluted to 300 ng/ml in hybridization solution with 2mg of sonicated salmon sperm DNA. Hybridization of the membrane with probe was done in a sealed bag at 64°C overnight. The membrane was washed 3 times with 250ml of low stringency wash buffer (see above) at 21°C and three times with high stringency wash buffer (see above) at 55°C. The membrane was then developed using the Pierce Chemiluminescent Nucleic Acid detection kit following manufacturer’s instructions (Thermo Scientific, Rockford IL). Membrane was exposed on film (Bioworld, Dublin OH) for 5 sec-30 min. The membrane was stripped by 30 min incubation in 0.4M NaOH at 45°C followed by 10 min in moderate stripping solution (200mM Tris-HCl pH 7.0, 0.1x SSC and 0.1% w/v SDS) at 21°C. The same membrane was re-hybridized with probe 1 as described above but using a hybridization temperature of 66°C.

### *T*. *gondii* replication assay

Confluent HFF monolayers grown on coverslips in 12- well plates were infected with 5x10^4^ parasites per well. Coverslips were fixed 12, 24, or 36 hr post-infection and processed for immunofluorescence as described above, using mouse anti-IMC1 MAb 45.36 and goat anti-mouse IgG conjugated to Alexa 488. All vacuoles in 100 randomly selected fields were scored and classified according to the number of parasites per vacuole.

### *T*. *gondii* invasion assay

1.5x10^5^ parasites were allowed to invade confluent HFF monolayers grown in each well of an 8-well chambered coverglass (Thermo Scientific) for 1 hr at 37°C. Loosely attached extracellular parasites were washed away with PBS and cells were fixed and processed for immunofluorescence as described above, using mouse anti-SAG1 IgG (Argene, Sherley NY) diluted 1:1000, followed by goat anti-mouse IgG conjugated to Alexa 546. After permeabilization with 0.25% v/v Triton X-100, samples were incubated sequentially with the same primary antibody followed by goat anti-mouse IgG conjugated to Alexa 488. Samples were imaged at 20X on a Nikon Eclipse TE300 epifluorescence microscope. All intracellular parasites in the well were counted and the continuity of the HFF cell monolayer across the entire well was confirmed by phase microscopy.

### Statistical analysis

Growth, differentiation, replication and invasion assays were analyzed using Student’s t-test or two-way ANOVA with GraphPad Prism5 (GraphPad Software, La Jollla CA).

## Results

### Compound 2 enhances *T*. *gondii* differentiation

We first tested whether Compound 2 could enhance parasite differentiation. For these experiments, we used a type I strain of *T*. *gondii* that shows a preference for homologous recombination because it lacks a key gene involved in non-homologous end joining (*KU80*) [38,39]. Because type I strains show low differentiation levels we also disrupted the Tg*UPRT* gene (S1 Fig) to interfere with the pyrimidine salvage pathway, thereby rendering these parasites more susceptible to differentiation under CO_2_ starvation conditions [36,40]. Parasites were allowed to invade a confluent host cell monolayer for 2 hr prior to addition of Compound 2 (or DMSO as vehicle) since Compound 2 inhibits parasite invasion of the host cell [24]. The percentage of parasite vacuoles that converted to cysts after a further 72 hr incubation under low CO_2_ conditions was assessed using fluorescently-conjugated *Dolichos biflorus* lectin (DL) which labels the sugars in the cyst wall [41] (Fig. 1B). As shown in Fig. 1C, Compound 2 treatment increased differentiation in both wild-type (non-disrupted Tg*UPRT*) and Δ*uprt* strains, compared to the vehicle-treated controls. A similar result was obtained with type II parasites (Prugniaud strain), despite these parasites showing higher basal levels of differentiation (S2 Fig).

### Compound 2 target profile in *T*. *gondii*

**Fig 2:**
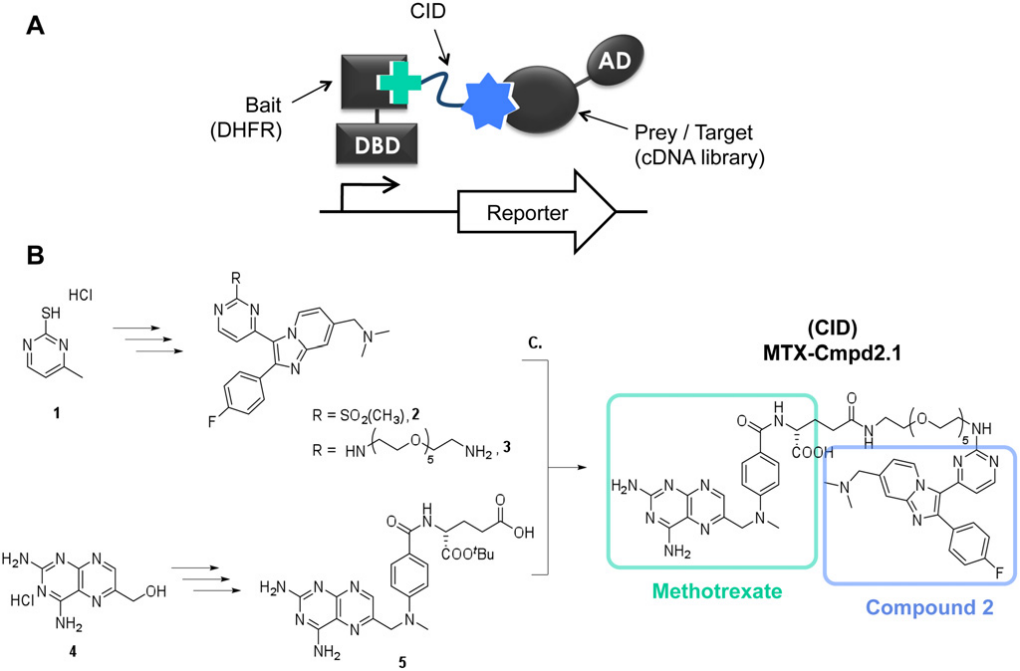
Y3H and CID synthesis. *A*. Schematic of the Y3H system showing the ternary complex between the CID and the two fusion proteins containing the activation and DNA-binding domains of the transcription factor (AD and DBD, respectively). The CID consists of: methotrexate (green cross), which binds to the DHFR-DBD fusion; a flexible linker unit (red); and the small molecule of interest (blue star), which binds to the target protein-AD fusion. *B*. Synthetic route to MTX-Cmpd2.1 (i) Compound 2-PEG linker fragment **3**; (ii) ^*t*^Butyl Methotrexate **5** and (iii) Peptide coupling and ^*t*^butyl deprotection to MTX-Cmpd2.1. For reagents and conditions, see S1 Methods.

As a first step in determining how Compound 2 enhances differentiation, we used yeast three-hybrid (Y3H) analysis [32,42–46] to examine the target profile of Compound 2 in *T*. *gondii*. Y3H is a modified version of yeast two-hybrid analysis in which the bait and prey/target proteins are brought together by a bridging molecule containing the small molecule of interest, thereby allowing identification of small molecule—protein interactions (Fig. 2A). The bivalent bridging molecule is referred to as a Chemical Inducer of Dimerization (CID). The bait and prey proteins are fused to the two functional domains of a transcription factor, so that the formation of a ternary complex consisting of bait, prey and CID leads to the activation of one or more reporter genes. Using this methodology, a cDNA library encoding parasite proteins can be screened for potential targets whose interaction with the CID leads to reporter activation.

The Compound 2-derived CID (MTX-Cmpd2.1) was prepared in three key steps (Fig. 2B). First, a published structure-activity relationship (SAR) study for Compound 2 suggested that the primary amino group was a suitable point for attachment of a linker, as substitution at this position resulted in retention of the compound’s inhibitory activity against its previously described targets [26]. Sulfone **2**, prepared in seven steps from **1** (see Scheme I in S1 Methods) was therefore converted to **3** through reaction with an *N*-Boc protected PEG linker [45] followed by subsequent *N*-Boc deprotection (Fig. 2B and Scheme II in S1 Methods). Second, the synthesis of ^*t*^butyl methotrexate **5** was achieved in good yield by *in situ* conversion of **4** to the corresponding bromide followed by reaction with 4-(methylamino)benzoic acid and coupling to a derivative of L-glutamic acid partially protected as the mono-*tert*-butyl ester (Fig. 2B and Scheme III in S1 Methods) [47]. Finally, TPTU-mediated coupling of **3** with **5** provided the ^*t*^butyl CID **S13**. Subsequent ester hydrolysis using TFA in the presence of thioanisole gave MTX-Cmpd2.1.

We used MTX-Cmpd2.1 to screen a *T*. *gondii* tachyzoite oligo(dT)-primed cDNA library and obtained 172 hits, *i*.*e*., yeast colonies that reproducibly grew on selective media differentially in the presence of CID (Table 1). Recovery and sequencing of the yeast plasmids revealed that the hits obtained did not encode any of the kinases previously described as targets of Compound 2 (TgPKG, TgCDPK1 and TgCK1α) [24]. Instead, a high percentage of the hits (81%) corresponded to TgME49_230180, which we named Tg*BRADIN* (BRAdyzoite Differentiation INhibitor) for reasons discussed below. Similar results were obtained in a second independent screen in which the hits were analyzed by colony hybridization for the presence of Tg*BRADIN* (discussed further below).

**Table 1:** Screen results with MTX-Cmpd2.1.

Number of hits that showed MTX-Cmpd2.1-dependent reporter activation	172
% Positive hits corresponding to TgME49_030180 (Tg*BRADIN*)	81
% Positive hits corresponding to Tg*CK1α*	0
% Positive hits corresponding to Tg*PKG*	0
% Positive hits corresponding to Tg*CDPK1*	0

Summary of the hits obtained in the screen with 5μM MTX-Cmpd2.1. The percentages indicated were obtained by sequence analysis of plasmids isolated from 36 of the colonies that showed MTX-Cmpd2.1-dependent reporter activation.

### Interaction between MTX-Cmpd2.1 and its predicted targets in the Y3H system

**Fig 3:**
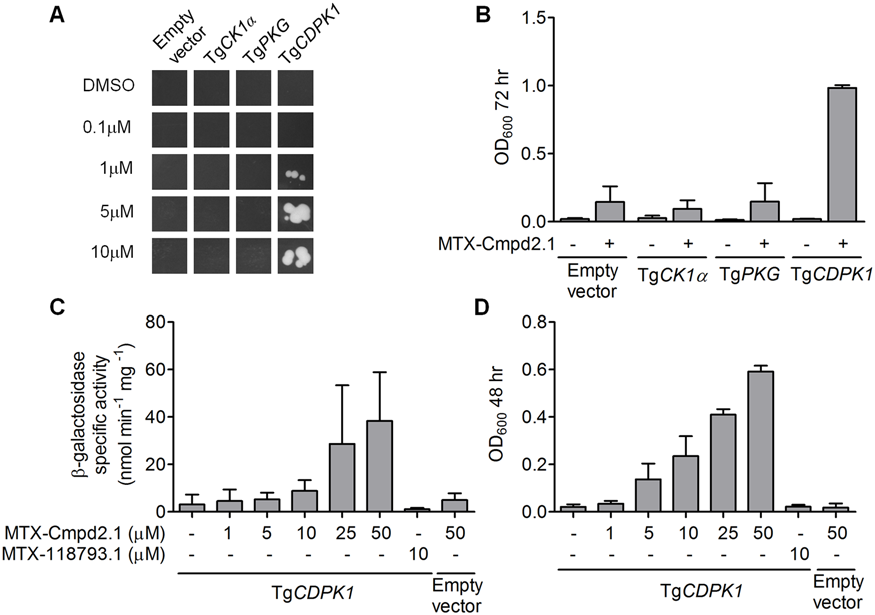
Interaction between MTX-Cmpd2.1 and the predicted targets of Compound 2 in the Y3H system. *A*. Y3H interaction between TgCK1α, TgPKG, TgCDPK1 and various concentrations of MTX-Cmpd2.1; representative images of the in-plate growth assay at 72 hr are shown. Yeast transformed with an empty pJG4–5 vector containing a stop codon immediately after the cloning site was used as a negative control. *B*. 72 hr liquid growth assays with either 10μM MTX-Cmpd2.1 or an equivalent amount of DMSO (mean ± SD, n = 3). *C and D*. Dose response analysis and specificity of the Y3H interaction of TgCDPK1 with MTX-Cmpd2.1 as measured by either (C) β-Galactosidase activity or (D) *LEU2*-mediated liquid growth in the presence of MTX-Cmpd2.1, MTX-118793.1 (an unrelated CID) or DMSO (mean ± SD, n ≥ 3).

The lack of interaction between Compound 2 and its predicted kinase targets in the Y3H screen was unexpected. PCR analysis demonstrated that sequences encoding full-length TgCK1α and the predicted compound-binding domain of TgPKG are present in the cDNA library, whereas the predicted compound-binding site in TgCDPK1 is not (data not shown). To test whether these three kinases are capable of interacting with MTX-Cmpd2.1 in a Y3H format, we cloned the entire coding sequence of each in frame with the B42 activation domain and tested it for interaction with the CID. Activation of the *LacZ* and *LEU2* reporter genes was evaluated in liquid growth and β-galactosidase assays. Of the three kinases, only TgCDPK1 interacted with MTX-Cmpd2.1 in the Y3H system (Fig. 3A and B; see also [46]). Reporter activation with TgCDPK1 was dose dependent for MTX-Cmpd2.1 concentrations in the micromolar range (Fig. 3C and D; [46]). Another methotrexate-containing CID (MTX-118793.1 [45]) did not activate any reporter genes, demonstrating that the interaction between TgCDPK1 and MTX-Cmpd2.1 is CID-specific (Fig. 3C and D).

While TgCDPK1 is capable of binding the Compound 2 portion of MTX-Cmpd2.1 and turning on the *LEU2* and *LacZ* reporter genes, it is not detectable within our cDNA library. This observation provided an opportunity to assess the sensitivity of our Y3H screening methods with respect to target copy number. We added Tg*CDPK1*-expressing yeast to the total yeast cDNA library in ratios ranging from 1:1,000 to 1:1,000,000. The pools were screened with MTX-Cmpd2.1 and the hits from the screens were analyzed by yeast colony hybridization to determine whether they contained Tg*CDPK1* or Tg*BRADIN* (S3 Fig). The data show that for a target compound pair such as Tg*CDPK1*-Compound 2 (IC_50_ = 0.7nM [24]), the target can be detected if it is represented in the library at a frequency of between 1:100,000 and 1:1,000,000. It therefore seems likely that TgCDPK1 did not appear as a hit in the initial Y3H screen due to under-representation in our cDNA library; the reason for the lack of interaction between MTX-Cmpd2.1 and its other two predicted kinase targets will be the subject of future investigation.

### TgBRADIN and its interaction with MTX-Cmpd2.1

**Fig 4:**
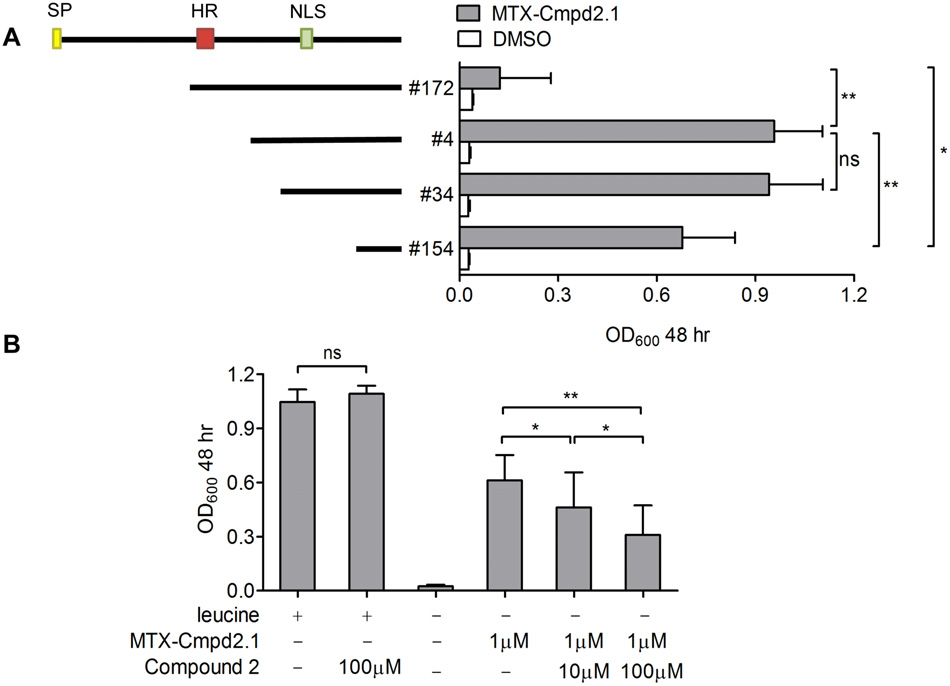
Y3H interaction between MTX-Cmpd2.1 and TgBRADIN. *A*. TgBRADIN (TgME49_230180) encodes a predicted N-terminal signal peptide (SP; yellow box), a central stretch of hydrophobic amino acids (HR; red box) and a predicted bipartite nuclear localization sequence (NLS; green box). Hits #172, 4, 34 and 154 were identified in the Y3H screen (starting at residues H173, T304, P324 and V398 and, respectively); coverage of the coding sequence of each is indicated by the black bars, and the corresponding activation of the *LEU2* reporter by 5μM MTX-Cmpd2.1 is shown on the right (mean ± SD, n = 3). The data were compared by paired Student’s t-test (*p < 0.015, **p < 0.01, ns = not significant,). *B*. Competition growth assay performed using hit #4 and MTX-Cmpd2.1 in the presence of various concentrations of Compound 2 as competitor. The first two columns correspond to growth in non-selective media and the following four correspond to the growth in media lacking leucine (mean ± SD, n = 3). The data were compared by one way ANOVA (*p < 0.05, **p < 0.01).

Tg*BRADIN* is a *T*. *gondii*-specific gene that has no identifiable orthologs, including in other apicomplexan parasites. The same gene was recently shown to encode a protein secreted into the host cell cytosol, possibly from a subset of the dense granules ([48]; see Discussion below). The protein contains a predicted N-terminal signal peptide, a second internal hydrophobic stretch and a bipartite nuclear localization signal that is likely responsible for trafficking of the secreted protein to the host cell nucleus (Fig. 4A; [48]).

The sequenced hits from the Y3H screen showed varying degrees of coverage of the Tg*BRADIN* coding sequence. Since the cDNA library was oligo(dT) primed, the coverage of Tg*BRADIN* included in all cases the 3’ end of the mRNA. We tested four hits from the screen with different degrees of coverage of the coding sequence (#172, 4, 34, 154; Fig. 4A) for their ability to interact with MTX-Cmpd2.1 in a *LEU2* reporter growth assay. As shown in Fig. 4A, the level of reporter gene activation correlates with the coverage of the open reading frame, although hit #172, which includes the internal hydrophobic stretch, shows very little reporter activation. The hydrophobic stretch may inhibit the Y3H interaction between TgBRADIN and MTX-Cmpd2.1 either because it disrupts the folding of the protein or interferes with trafficking into the yeast nucleus. These data demonstrate that the N-terminal half of TgBRADIN is dispensible for its Y3H interaction with MTX-Cmpd2.1.

Fig. 4B shows that the interaction between MTX-Cmpd2.1 and TgBRADIN can be competed by an excess of Compound 2. This observation, together with the lack of interaction of MTX-118793.1 with TgBRADIN (Fig. 3C and D), confirms that the Y3H interaction of TgBRADIN and MTX-Cmpd2.1 involves the Compound 2 moiety, rather than some other part of the CID.

### Tg*BRADIN* is alternatively spliced and its Y3H interaction with Compound 2 is isoform-specific

**Fig 5:**
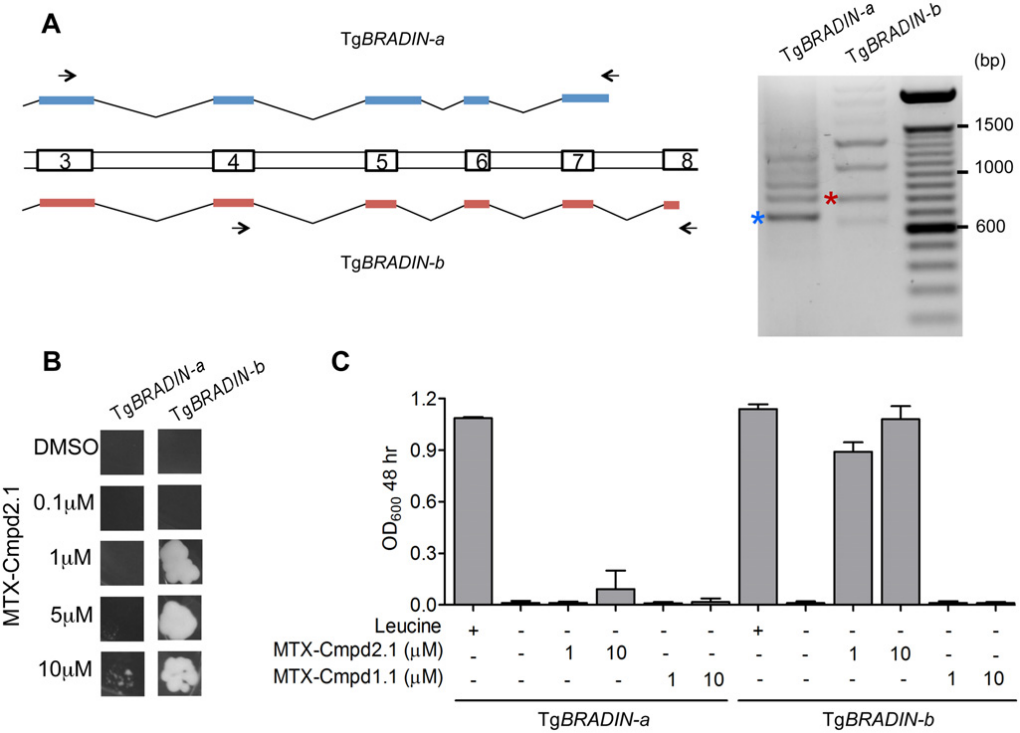
The TgBRADIN Y3H interaction with Compound 2 is isoform-specific. *A*. Schematic showing exons 3–8 from two of the Tg*BRADIN* isoforms generated by alternative splicing of Tg*BRADIN* (see S1A Fig for schematic of the entire gene). Two PCRs are shown on the right using primers P7 and P8 for Tg*BRADIN-a* and P17 and P18 for Tg*BRADIN-b* (arrows). The bands marked with the asterisks correspond to the splicing pattern of either Tg*BRADIN-a* (blue) or Tg*BRADIN-b* (red, hit #4 from the Y3H screen) as determined by sequencing of the PCR products. *B*. The C-terminal half of the coding sequence corresponding to either Tg*BRADIN-a* or Tg*BRADIN-b* was tested in an in-plate growth assay (5 days at 30°C) with MTX-Cmpd2.1 at the concentrations shown. *C*. The same yeast expressing Tg*BRADIN-a* or Tg*BRADIN-b* were used in a liquid growth assay with MTX-Cmpd2.1 or MTX-Cmpd1.1 (a Compound 1-derived CID) (mean ± SD, n = 3).

During attempts to amplify the Tg*BRADIN* gene from cDNA, we discovered that multiple distinct mRNA transcripts are generated from the Tg*BRADIN* locus and that these transcripts are created by alternative splicing. We focused on the portions of the transcripts that encode the C-terminal half of Tg*BRADIN* (exons 3–8, see S1 Fig for gene schematic), the region required for a positive Y3H interaction with the Compound 2-based CID. We PCR amplified this portion of the predicted gene from cDNA using two sets of primers (Fig. 5A) and sequenced several isoforms. Two isoforms were chosen that encode proteins with different C-terminal ends, Tg*BRADIN-a* and Tg*BRADIN-b*. Tg*BRADIN*-a, which encodes a fragment that extends from the central hydrophobic stretch to the C-terminus, was cloned in frame with the activation domain in the target Y3H vector. Hit #4 from the Y3H screen (Fig. 4A) was used as a representative Tg*BRADIN-b* sequence. Each was tested for its ability to interact with MTX-Cmpd2.1 by Y3H. The TgBRADIN-b isoform showed much more sensitive compound-dependent transcription of the *LEU2* reporter gene than TgBRADIN-a (Fig. 5B and C). In agreement with this result, none of the hits sequenced in the original Y3H screen with MTX-Cmpd2.1 contained the Tg*BRADIN-a* splicing pattern at the 3’ end. These results show that different naturally-occurring isoforms of TgBRADIN interact differentially with Compound 2 in the Y3H format.

We also explored whether reporter activation with TgBRADIN-b was specific for Compound 2 or if it could also occur with the related small molecule, Compound 1. As shown in Fig. 5C, while TgBRADIN-b shows a strong Y3H interaction with MTX-Cmpd2.1, it shows no detectable interaction with a CID based on Compound 1 (MTX-Cmpd1.1).

### Parasites lacking Tg*BRADIN* show a differentiation phenotype

**Fig 6:**
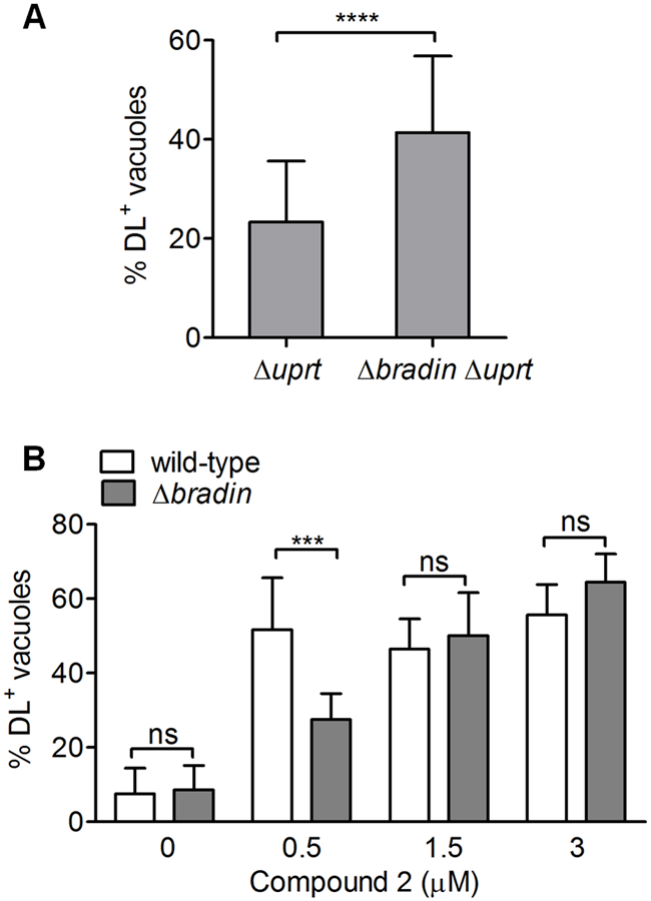
Parasites lacking Tg*BRADIN* show increased differentiation and reduced sensitivity to Compound 2. *A*. Differentiation of Δ*uprt* and Δ*bradin* Δ*uprt* strains under CO_2_ starvation was measured either by staining with *Dolichos biflorus* lectin (DL; mean ± SD, n = 21) or by immunofluorescence with anti-BAG1 (BAG1; mean ± SD, n = 3). The data were compared by paired Student’s t-test (*p < 0.05, ****p < 0.0001). *B*. Differentiation assay comparing parental wild-type and Δ*bradin* strains treated with either Compound 2 or an equivalent volume of DMSO for 72 hr under CO_2_ starvation conditions (mean ± SD, n ≥ 4). The data were compared by two-way ANOVA (***p < 0.001, ns = not significant).

The observation that Compound 2 both enhances bradyzoite differentiation and interacts with TgBRADIN in Y3H assays led us to hypothesize that Tg*BRADIN* functions in differentiation. To test this hypothesis, we deleted the Tg*BRADIN* gene in the wild-type strain (S2 Table). Diagnostic PCRs confirmed that the Tg*BRADIN* locus was disrupted and Southern blotting demonstrated that the deletion cassette integrated only once in the genome, at the Tg*BRADIN* locus (S1 Fig). We then disrupted the Tg*UPRT* gene in this background, as described above, in order to test the ability of the Tg*BRADIN* knockout parasites to differentiate under low CO_2_ conditions. The Δ*bradin*Δ*uprt* parasites showed a significant enhancement of differentiation upon CO_2_ starvation compared to parasites expressing TgBRADIN, as assayed either by binding of *Dolichos biflorus* lectin or by expression of the bradyzoite-specific marker BAG1 (Fig. 6A).

When parasites are induced to differentiate into bradyzoites their cell cycle slows and they enter into a lateS/G2 phase, suggesting a connection between cell cycle regulation and initiation of the differentiation program [49,50]. A mutant parasite that differentiates better than wild type might therefore replicate more slowly. We tested this hypothesis by evaluating the replication rate of wild-type and Δ*bradin* (Tg*UPRT* ^+^) parasites under normal tachyzoite growth conditions, and saw no differences in the number of parasites per vacuole at any time point during the assay (S4 Fig). Generation of the Δ*bradin* parasite strain also allowed us to test whether TgBRADIN plays a role in invasion. The Δ*bradin* parasites showed no defect in invasion compared to wild type (S5 Fig) suggesting that Compound 2’s effect on differentiation and its previously reported effect on invasion [24] likely occur through different mechanisms.

If TgBRADIN plays a role in Compound 2’s ability to enhance differentiation, then the Δ*bradin* parasites should be less sensitive to Compound 2 treatment. To test this hypothesis, we treated wild-type and Δ*bradin* parasites with varying concentrations of Compound 2 and assessed their ability to differentiate under CO_2_ starvation conditions. We used Tg*UPRT* ^+^ strains in this case to maximize the difference between treatments since the Δ*uprt* strains show a higher basal level of differentiation (Fig. 1C). At Compound 2 concentrations of 1.5μM or higher, there was no difference in differentiation between wild-type and Δ*bradin* parasites, but when the concentration of Compound 2 was decreased to 0.5μM the Δ*bradin* parasites showed half the induction of wild type (Fig. 6B). These data demonstrate that a portion of the differentiation enhancement caused by Compound 2 is indeed mediated by Tg*BRADIN*, and suggest that other as yet unidentified target(s) of Compound 2 also contribute to the process.

## Discussion

The mechanisms and pathways underlying *T*. *gondii* differentiation remain poorly understood, despite numerous studies aimed at identifying differentiation effector and regulator genes [8–10,12–16,51]. An important recent advance was the identification of *T*. *gondii* transcription factors containing AP2 DNA binding domains and the demonstration that some of these transcription factors regulate the tachyzoite-to-bradyzoite/tissue cyst transition [17,18]. For example, overexpression of AP2IX-9 represses bradyzoite induction, and disruption of AP2IX-9 increases cyst formation [17]. AP2IX-9 therefore functions as a repressor of tachyzoite-to-bradyzoite differentiation, and likely acts by binding to *cis*-regulatory DNA elements that control stage-specific gene expression [17].

TgBRADIN and the serine protease inhibitor, TgPI [51], are two other tachyzoite proteins that appear to function as negative regulators of *T*. *gondii* differentiation. As with Tg*BRADIN*, parasites in which Tg*PI* is disrupted show an increased rate of *in vitro* differentiation, and the expression data available in ToxoDB [52] show that transcription of both Tg*PI* and Tg*BRADIN* decreases shortly after tachyzoites are induced to differentiate into bradyzoites. The expression of Tg*BRADIN* is in fact highest in the oocyst stage, intermediate in tachyzoites and lowest in bradyzoites. Oocysts are produced within the intestine of the cat and must differentiate into sporozoites prior to infecting a new host; it will therefore be of interest to determine whether Tg*BRADIN* plays any role in regulating the oocyst-to-sporozoite transition.

While this manuscript was under review, another group independently published a study on Tg*BRADIN* (which they named *GRA24* [48]), and their results suggest an intriguing potential mechanism by which Tg*BRADIN/GRA24* and Compound 2 might affect differentiation. In brief, these authors showed that TgBRADIN/GRA24 is secreted by the intracellular parasite into the host cell cytosol, where it associates directly with host p38α MAP kinase (p38α MAPK). Binding of TgBRADIN/GRA24 to p38α MAPK results in an unusually persistent autophosphorylation/activation of the kinase and its translocation to the host cell nucleus. Within the nucleus, the activated kinase upregulates several important host cell transcription factors and alters the expression of a number of genes, including those that control proinflammatory cytokine and chemokine production [48]. It is possible that TgBRADIN/GRA24 regulates parasite differentiation through similar p38α MAPK-mediated changes in host cell gene expression; further studies will be required to test this hypothesis and to identify the specific genes involved. There is precedent for a host cell gene mediating the differentiation-enhancing activity of a small molecule: the ability of Compound 1 to enhance bradyzoite formation depends, in part, on the host cell cycle regulator *CDA-1* [22].

How would Compound 2 enhance differentiation in this model? Because Compound 2 is an ATP analog and well-established kinase inhibitor [23,24], we hypothesize that it binds to p38α MAPK rather than directly to TgBRADIN/GRA24 (which has no apparent ATP-binding motif). This would be consistent with our inability to show direct binding of Compound 2 to recombinantly expressed TgBRADIN/GRA24 by a variety of biochemical techniques (AVO and GEW, unpublished data). However, if Compound 2 binds to p38α MAPK and not TgBRADIN/GRA24, why did we so consistently recover TgBRADIN/GRA24 in our Y3H screens with MTX-Cmpd2.1? We propose that the TgBRADIN/GRA24-activation domain fusions in our cDNA library bind to yeast p38α MAPK (also known as HOG1), which in turn binds to the Compound 2 portion of the CID, ultimately forming a (DNA binding domain-DHFR)—(MTX-Cmpd2.1)—(p38α MAPK)—(TgBRADIN/GRA24-activation domain) quaternary complex that activates reporter gene expression (see schematic, S6 Fig). This model is consistent with our observation that the C-terminal half of TgBRADIN/GRA24, *i*.*e*., the portion that contains the KIM/D motifs required for binding to p38α MAPK [48], is sufficient for a positive Y3H interaction with MTX-Cmpd2.1 (Fig. 4A). While the actual formation of such a quaternary complex will require biochemical confirmation, these results raise the possibility that Y3H can be used to identify both direct *and* indirect binding partners of small molecules of interest. At the same time, they sound a cautionary note about how Y3H results should be interpreted and hits validated.

One intriguing feature of the Tg*BRADIN/GRA24* gene is the large number of splice variants expressed, likely beyond the two isoforms previously described [48]). It is, in fact, among the most highly alternatively spliced genes in the transcriptome (B. Thompson and M. Matrajt, personal communication). This suggests either high functional versatility or complex regulation of Tg*BRADIN/GRA24* expression. Tg*PI* also undergoes alternative splicing, into two isoforms, neither of which alone can fully reverse the enhanced differentiation observed in parasites with the Tg*PI* gene disrupted. Alternative splicing as a regulatory mechanism has been understudied in *T*. *gondii*. Recent analyses suggest that a large number of *T*. *gondii* transcripts are in fact alternatively spliced ([53] and http://www.toxodb.org), introducing another level of variability and dynamics to the regulation of gene expression in *T*. *gondii* and expanding the total number of protein isoforms the parasite is capable of producing. A complete catalog of all the biologically relevant splicing isoforms generated by Tg*BRADIN/GRA24*, their stage specificities, and their biological functions will be the subject of future investigation.

The Tg*BRADIN/GRA24* knockout parasites were only partially resistant to the effects of Compound 2, suggesting either that compensatory parasite mutations developed during generation or growth of the knockout or, more likely, that other direct or indirect targets of Compound 2 are also involved. Tg*PKG*, Tg*CDPK1* and/or Tg*CK1α*, all known targets of Compound 2 [24], might have an undiscovered role in differentiation, synergizing with Tg*BRADIN/GRA24* during this process. Given the similarities between Compound 2 and Compound 1, the unidentified target of Compound 1 in the host cell that mediates its differentiation-enhancing activity through *CDA-1* [22] represents another potential target of Compound 2 that could synergize with Tg*BRADIN/GRA24* to produce the differentiation phenotype observed. A systematic analysis of these potential targets and additional Y3H screens with alternative Compound 2-based CIDs and different cDNA libraries (including host cell cDNA libraries) will ultimately help to establish the complete target profile of Compound 2 in *T*. *gondii*-infected cells.

Differentiation is essential for *T*. *gondii*’s successful transmission between intermediate hosts. Most acute infections in humans are acquired by ingestion of undercooked meat of animals containing tissue cysts or oocyst-contaminated food and water [54]. The infection develops into the chronic phase, which is generally not a threat to human health. However, if the host is immunocompromised, the recrudescence of a chronic infection, during which latent bradyzoites differentiate to tachyzoites, can be life-threatening [2,3]. As a consequence, chemotherapeutics that either eliminate tissue cysts or prevent their formation would be clinically useful. While there are currently no drugs available for this purpose, our results and those of others [7,13,22,55,56] demonstrate clearly that small molecules are capable of affecting the pathways that regulate differentiation. High-throughput small molecule screening for compounds that affect differentiation may therefore provide a new approach to discovering drugs that block cyst formation or reactivation, and Y3H will provide a complementary means to determine the target profile of these bioactive compounds.

## Supporting Information

S1 Fig Generation of the Δ*bradin* strain. *A*. Schematic showing the strategy used to obtain the Δ*bradin* parasite line. Empty boxes represent predicted exons (numbered) in the Tg*BRADIN* locus. Hatched areas flanking the *TgBRADIN* gene were used for homologous recombination of the phleomycin resistance cassette (ble^R^) into the locus. Both the parental wild-type and Δ*bradin* parasite lines (parental clones 1 and 2 and Δ*bradin* clones 3 and 4) were transfected to disrupt the Tg*URPT* locus. S: *Ssp*I restriction sites. *B*. PCRs were performed on gDNA of isolated parasite clones, amplifying the regions indicated in panel A. Expected amplicon size of PCR A: 0.9kb and PCR B: 0.8kb. PCR C, performed as described previously [36], was used to confirm the disruption of the Tg*UPRT* locus (Δ*uprt*: 0.5kb; Tg*UPRT*: 1.5kb). *C*. Ethidium bromide stained gel of SspI digested genomic DNA from wild-type (clone 1) and Δ*bradin* (clone 3) parasites. Southern blot showing the hybridization of probes 1 and 2 on the digested DNA; the expected 2.1 and 2.5kb restriction fragments (see panel A) are indicated by the orange and green arrowheads, respectively.
(TIF)

S2 Fig Compound 2 enhances differentiation in Type II parasites. Percentage of *Dolichos* lectin-positive vacuoles (DL+) in samples treated with either DMSO or Compound 2 (3μM) for 72 hr under CO_2_ starvation conditions (mean ± SD, n = 3). The data were compared by paired Student’s t-test (*p < 0.05).
(TIF)

S3 Fig Determining the sensitivity of Tg*CDPK1* detection in the Y3H screening format with MTX-Cmpd2.1. *A*. A series of screens were undertaken using the unmodified cDNA library and the same library spiked with varying ratios of yeast carrying the plasmid pJG4–5 containing the Tg*CDPK1* coding sequence. Forty colonies from each screen were analyzed by colony hybridization to determine the number that carried a plasmid encoding either Tg*CDPK1* or Tg*BRADIN*. The black dots correspond to the hybridization signal using a probe directed against either Tg*CDPK1* or Tg*BRADIN*, as indicated on the left. *B*. Graphical representation of the results from panel A.
(TIF)

S4 Fig Parasites lacking Tg*BRADIN* show no replication defect. Replication assay comparing wild-type and Δ*bradin* parasites. Number of parasites per vacuole was determined at 12, 24 and 36 hrs post-infection. 100 fields were counted at each timepoint, with two replicates per experiment (mean ± SD shown, n = 3). No significant differences were found by Student’s t-test at any time point.
(TIF)

S5 Fig Parasites lacking Tg*BRADIN* show no invasion defect. Invasion assay comparing wild-type and Δ*bradin* parasites (mean ± SD, n = 2). No significant differences were found between the strains by Student’s t-test.
(TIF)

S6 Fig Schematic of a hypothetical quaternary complex between DHFR, MTX-Cmpd2.1, yeast p38α MAPK and TgBRADIN/GRA24 that supports Y3H reporter gene activation. Yeast p38α MAPK (orange) is predicted bind both to the Compound 2 portion of MTX-Cmpd2.1 (blue) and to the C-terminal KIM/D motifs [48] of the TgBRADIN/GRA24-AD fusion (red). Simultaneous binding of the MTX portion of the CID (green) to the DHFR-DBD fusion (purple), reconstitutes the transcription factor and activates the reporter gene. AD = Activation domain, DBD = DNA binding domain.
(TIF)

S1 Methods Supplementary Chemical Synthesis Methods. (DOCX)

S1 Table Primers used in this study. (DOC)

S2 Table *Toxoplasma gondii* strains used in this study. (DOC)
