# Supplementary figures and images for: Yeast Three-Hybrid Screen Identifies TgBRADIN/GRA24 as a Negative Regulator of Toxoplasma gondii Bradyzoite Differentiation
Source: PLoS One. 2015 Mar 19;10(3):e0120331. doi: 10.1371/journal.pone.0120331 (PMC4366382; doi:10.1371/journal.pone.0120331)

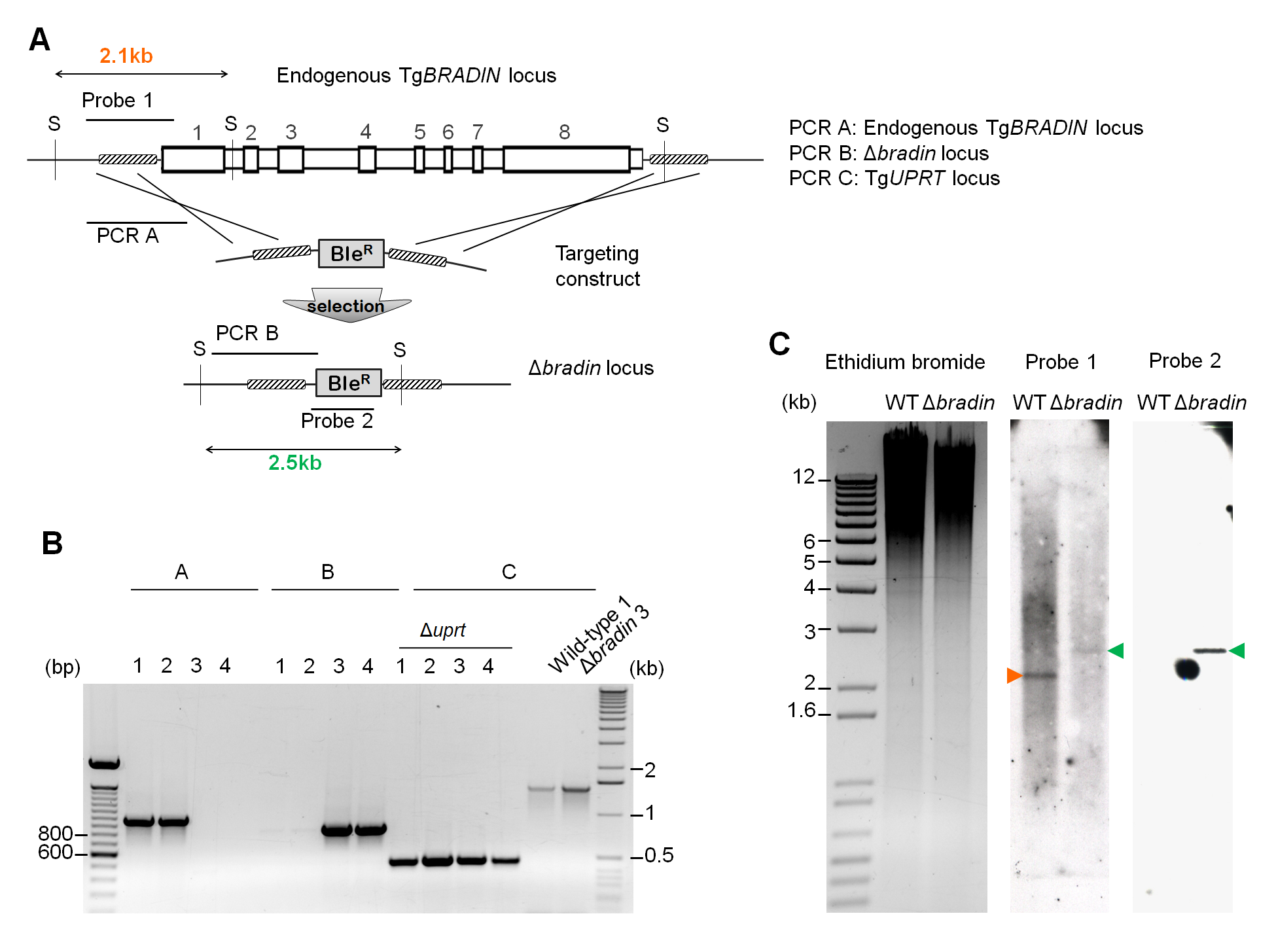

Supplement: S1 Fig — A. Schematic showing the strategy used to obtain the Δbradin parasite line. Empty boxes represent predicted exons (numbered) in the TgBRADIN locus. Hatched areas flanking the TgBRADIN gene were used for homologous recombination of the phleomycin resistance cassette (bleR) into the locus. Both the parental wild-type and Δbradin parasite lines (parental clones 1 and 2 and Δbradin clones 3 and 4) were transfected to disrupt the TgURPT locus. S: SspI restriction sites. B. PCRs were performed on gDNA of isolated parasite clones, amplifying the regions indicated in panel A. Expected amplicon size of PCR A: 0.9kb and PCR B: 0.8kb. PCR C, performed as described previously [36], was used to confirm the disruption of the TgUPRT locus (Δuprt: 0.5kb; TgUPRT: 1.5kb). C. Ethidium bromide stained gel of SspI digested genomic DNA from wild-type (clone 1) and Δbradin (clone 3) parasites. Southern blot showing the hybridization of probes 1 and 2 on the digested DNA; the expected 2.1 and 2.5kb restriction fragments (see panel A) are indicated by the orange and green arrowheads, respectively. (TIF) [file pone.0120331.s001.tif]

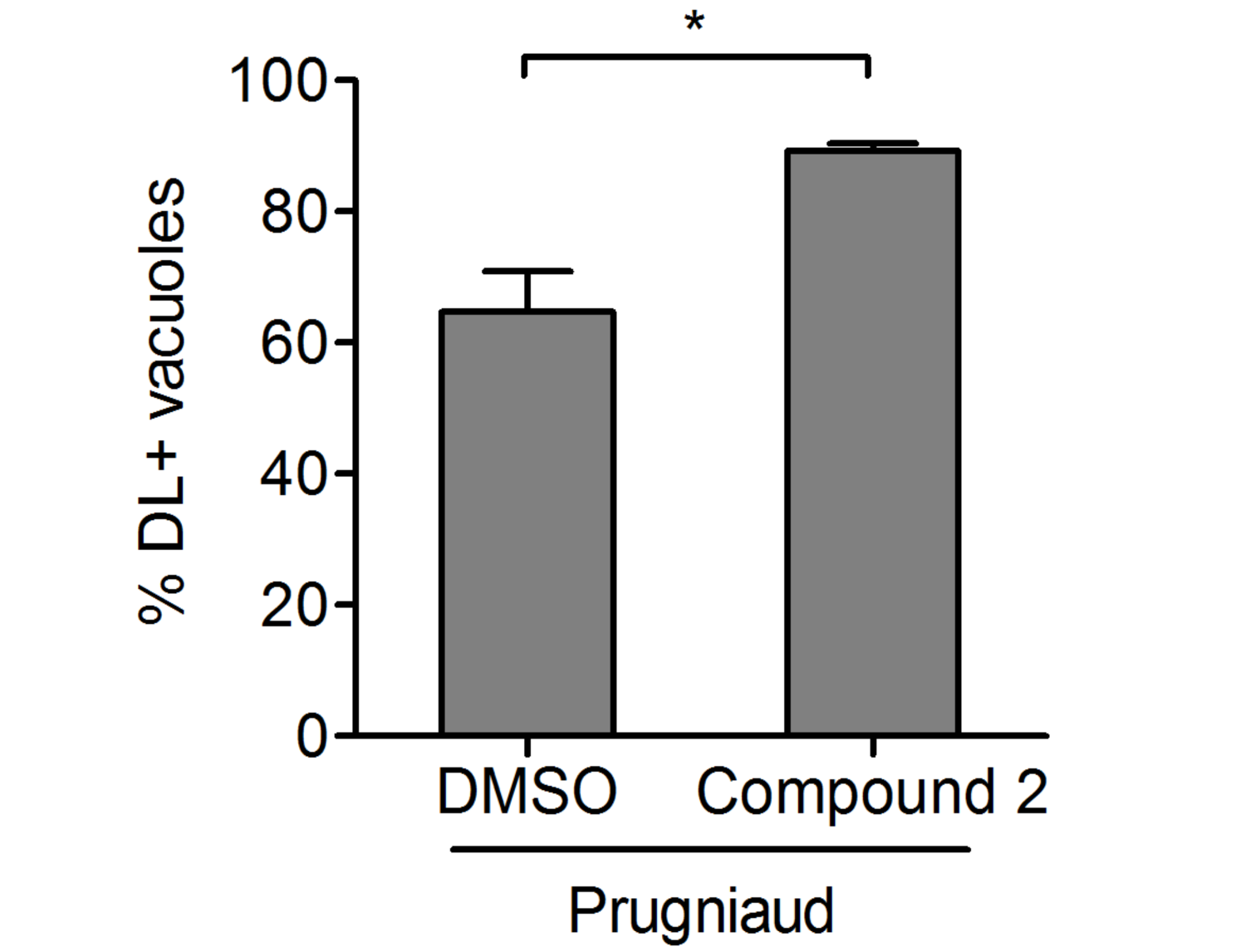

Supplement: S2 Fig — Percentage of Dolichos lectin-positive vacuoles (DL+) in samples treated with either DMSO or Compound 2 (3μM) for 72 hr under CO2 starvation conditions (mean ± SD, n = 3). The data were compared by paired Student’s t-test (*p < 0.05). (TIF) [file pone.0120331.s002.tif]

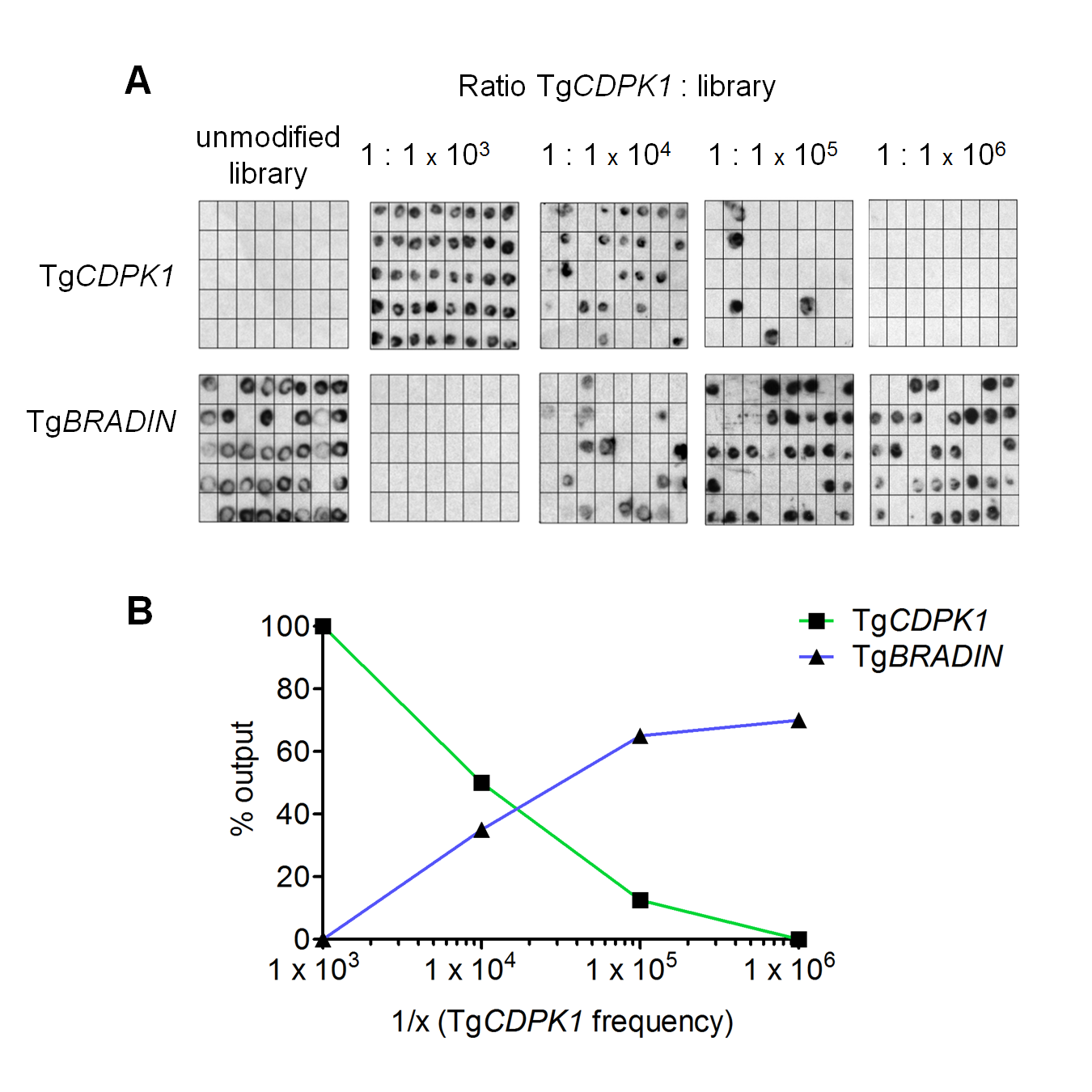

Supplement: S3 Fig — A. A series of screens were undertaken using the unmodified cDNA library and the same library spiked with varying ratios of yeast carrying the plasmid pJG4–5 containing the TgCDPK1 coding sequence. Forty colonies from each screen were analyzed by colony hybridization to determine the number that carried a plasmid encoding either TgCDPK1 or TgBRADIN. The black dots correspond to the hybridization signal using a probe directed against either TgCDPK1 or TgBRADIN, as indicated on the left. B. Graphical representation of the results from panel A. (TIF) [file pone.0120331.s003.tif]

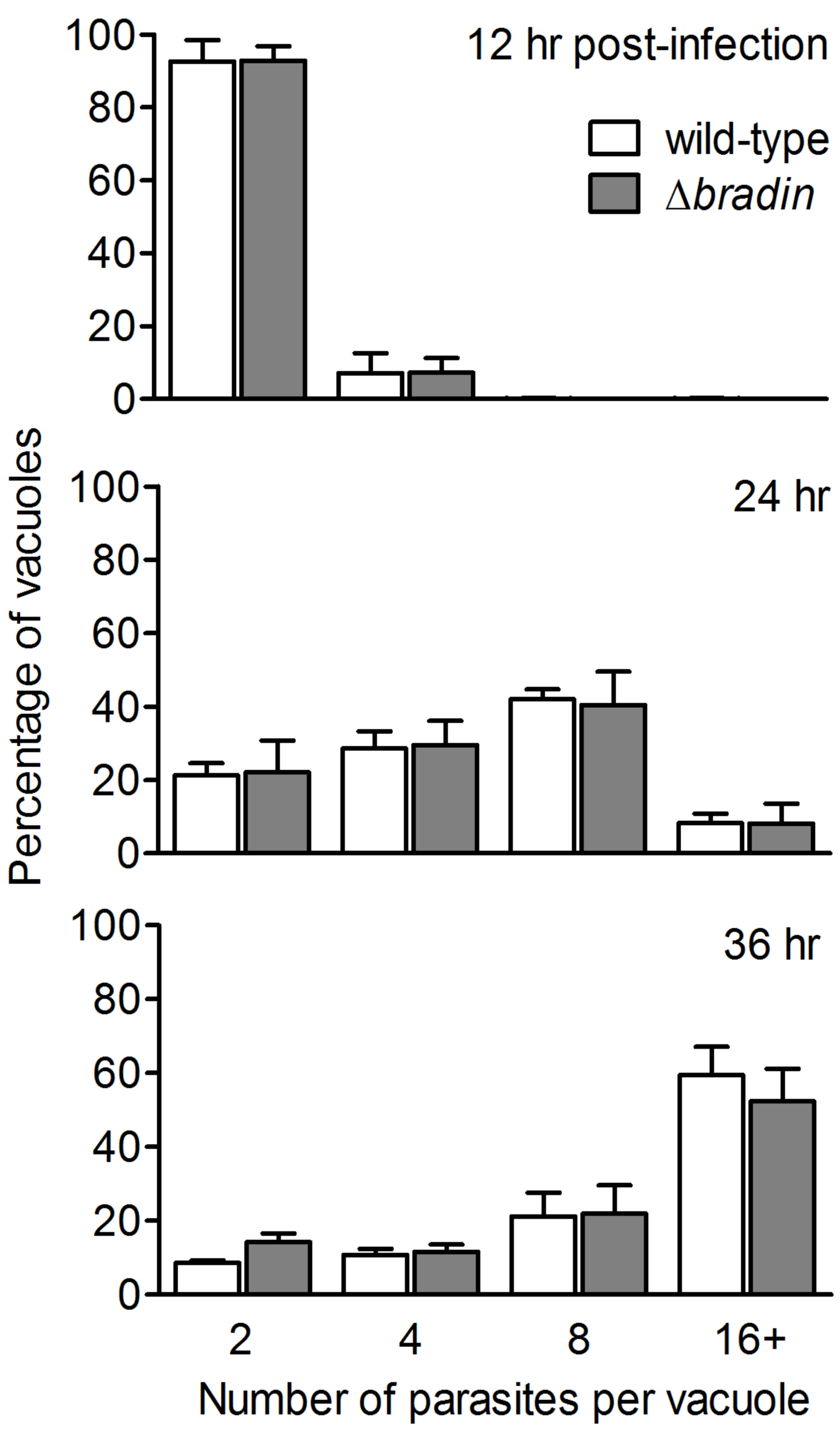

Supplement: S4 Fig — Replication assay comparing wild-type and Δbradin parasites. Number of parasites per vacuole was determined at 12, 24 and 36 hrs post-infection. 100 fields were counted at each timepoint, with two replicates per experiment (mean ± SD shown, n = 3). No significant differences were found by Student’s t-test at any time point. (TIF) [file pone.0120331.s004.tif]

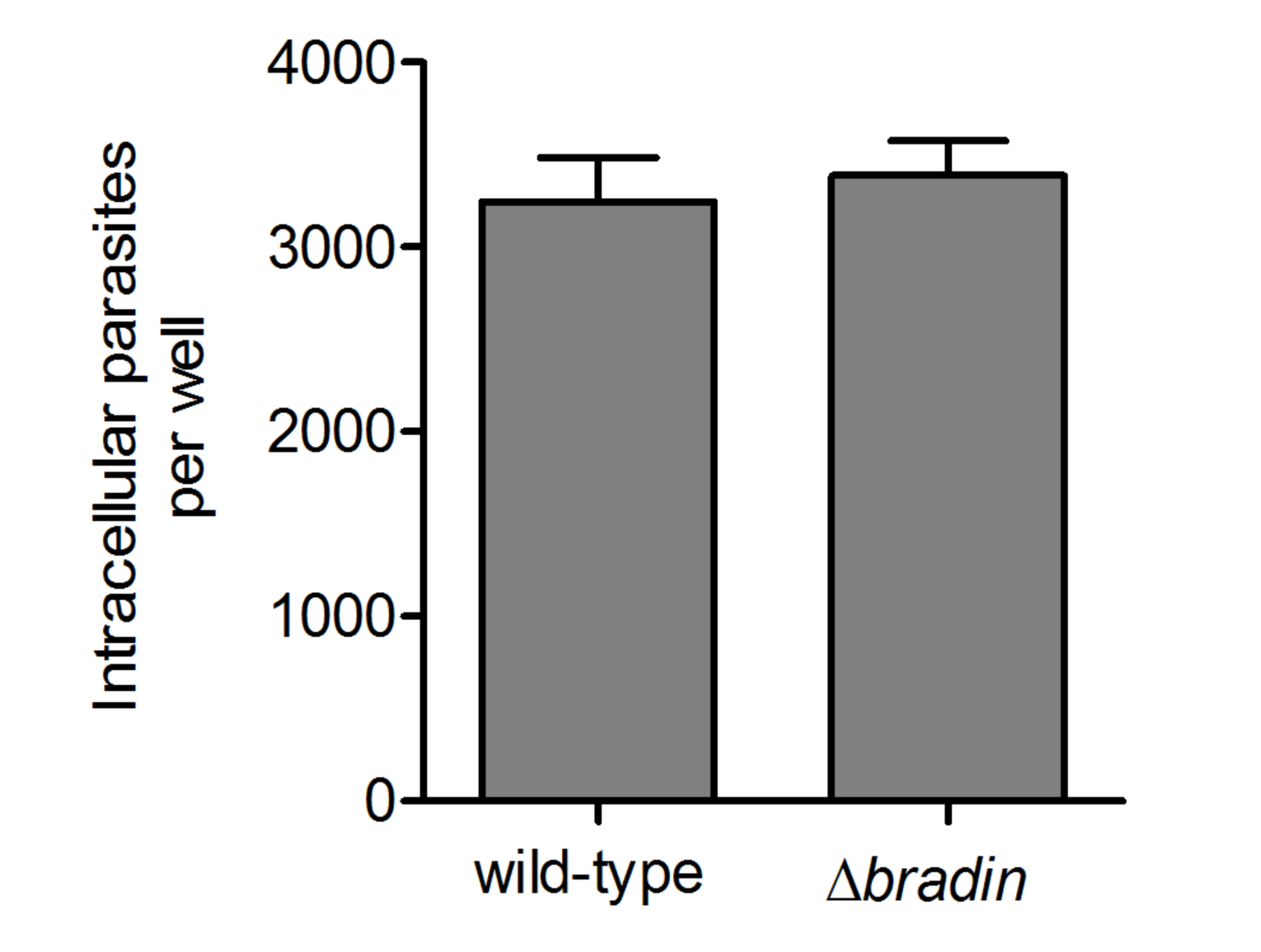

Supplement: S5 Fig — Invasion assay comparing wild-type and Δbradin parasites (mean ± SD, n = 2). No significant differences were found between the strains by Student’s t-test. (TIF) [file pone.0120331.s005.tif]

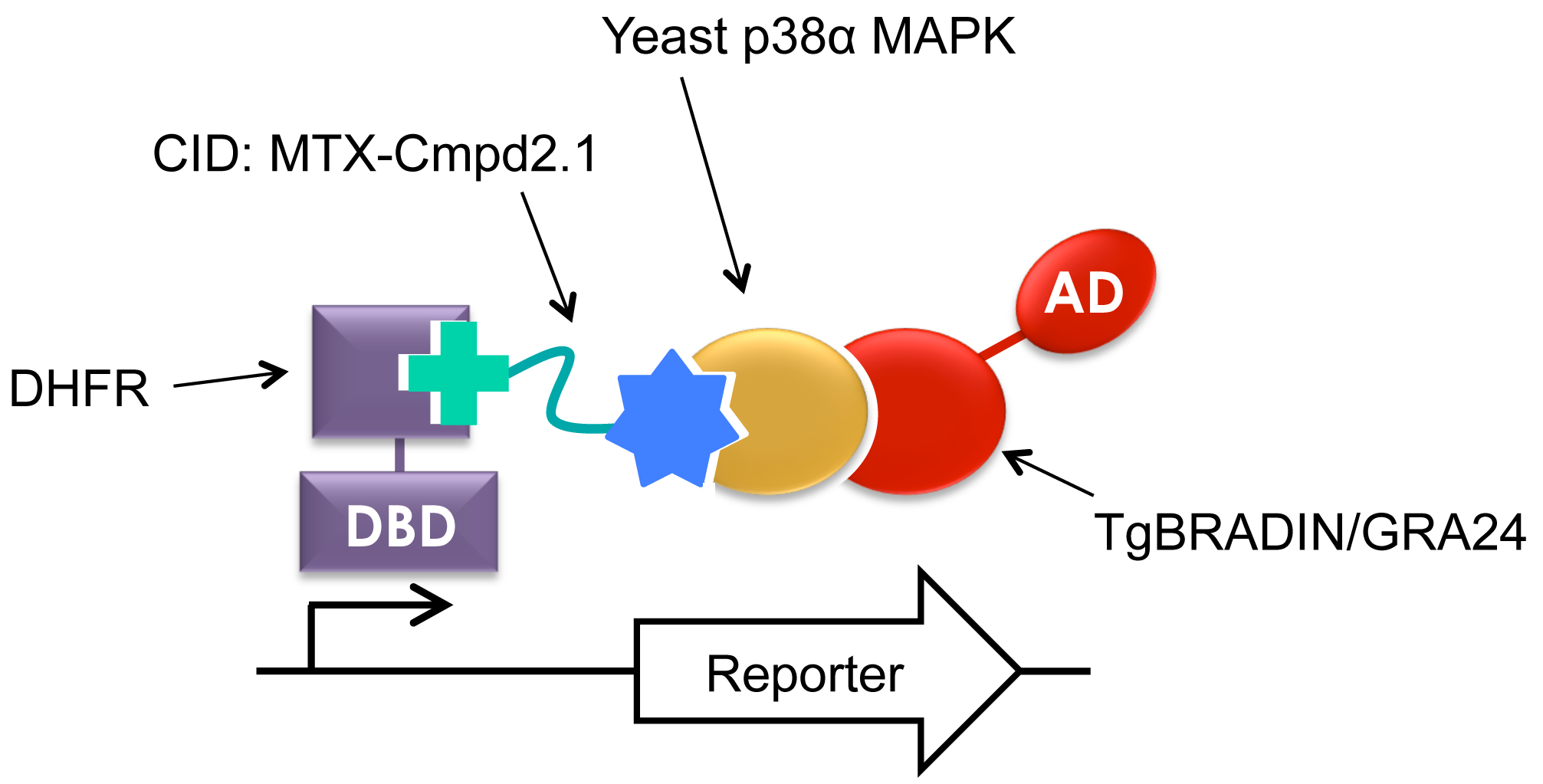

Supplement: S6 Fig — Yeast p38α MAPK (orange) is predicted bind both to the Compound 2 portion of MTX-Cmpd2.1 (blue) and to the C-terminal KIM/D motifs [48] of the TgBRADIN/GRA24-AD fusion (red). Simultaneous binding of the MTX portion of the CID (green) to the DHFR-DBD fusion (purple), reconstitutes the transcription factor and activates the reporter gene. AD = Activation domain, DBD = DNA binding domain. (TIF) [file pone.0120331.s006.tif]
