# Supplementary material for: Yeast Three-Hybrid Screen Identifies TgBRADIN/GRA24 as a Negative Regulator of Toxoplasma gondii Bradyzoite Differentiation
Source: PLoS One. 2015 Mar 19;10(3):e0120331. doi: 10.1371/journal.pone.0120331 (PMC4366382; doi:10.1371/journal.pone.0120331)
Supplement: S1 Methods — (DOCX) [file pone.0120331.s007.docx]

**Supplementary Chemical Synthesis Methods**

All chemicals and solvents were purchased from Aldrich (Gillingham, UK), Alfa Aesar (Ward Hill, MA, USA ) or Apollo Scientific (Chesire, UK) and used without further purification. All reactions were carried out under a positive pressure of nitrogen or in oven-dried glassware. Triethylamine and diisopropylamine were dried by distillation from KOH and stored over KOH. Anhydrous tetrahydrofuran (THF), dichloromethane (DCM) and hexanes were obtained from a solvent purification system (MBraun, SPS-800).

Thin layer chromatography (TLC) analysis was performed using glass plates coated with silica gel (with fluorescent indicator UV_254_). Developed plates were air dried and analysed under a UV lamp (254/365 nm). Flash chromatography was performed using silica gel (40-63µm, Fluorochem).

Infrared spectra were recorded on a Perkin Elmer Spectrum GX FT-IR spectrometer using either thin films on NaCl plates (NaCl) or KBr discs (KBr) as stated. Absorption maxima are reported as wavenumbers (cm^-1^).

Low resolution (LR) and high resolution (HR) electrospray mass spectral (ES-MS) analyses were acquired by electrospray ionisation (ESI). These were acquired within the School of Chemistry, University of St Andrews.

Nuclear magnetic resonance (NMR) spectra were acquired at room temperature on either a Bruker Avance 300 (^1^H, 300.1 MHz; ^13^C, 75.5 MHz), Bruker Avance II 400 (^1^H, 400.1 MHz; ^13^C, 100.6 MHz), Bruker Avance 500 (^1^H, 500 MHz; ^13^C, 125.7 MHz) or Bruker Avance III 500 (^1^H, 500.1 MHz, ^13^C, 125.7 MHz) spectrometers and in the deuterated solvent stated. All NMR spectra were acquired using the deuterated solvent as the lock. Coupling constants (*J*) are quoted in Hz and are recorded to the nearest 0.1 Hz. The following abbreviations are used; s, singlet; d, doublet; dd, doublet of doublets; ddd, doublet of doublets of doublets; dt, doublet of triplets; t, triplet; tdd, triplet of a doublets of a doublets ; m, multiplet; q, quartet; qt, quintet; and br, broad.

Chemical shifts are expressed as δ in units of ppm. ^13^C NMR spectra were recorded under the same conditions and solvents using the PENDANT sequence mode. Data processing was carried out using the TOPSPIN 2 NMR program (Bruker UK Ltd).

1. **Synthesis of Compound 2**

The synthesis of Compound 2 started from 4-methylpyrimidine-2-thiol hydrochloride (**1**) *via* a modified version of the procedure reported by Scribner et al. [1] In brief, **1** was S-methylated and coupled to methyl-4-fluorobenzoate to give a mixture of the keto and enol tautomers **S2** and **S3** (Scheme S1). Subsequent bromination of **S2** and **S3** using NBS gave **S4**, which was reacted with (2-aminopyridin-4-yl)methanol **S6**) to give **S7**. Oxidation of the sulfide group in **S7** to the corresponding sulfone **S8** was followed by mesylation of the primary alcohol and displacement by dimethylamine to give sulfone **2**. This modified procedure was carried out on a large scale and in relatively good yield. **2** was converted to Compound **2** as shown.

**Scheme S1:** Synthetic route to Compound 2

**1-(4-Fluorophenyl)-2-(2-(methylthio)pyrimidin-4-yl)ethanone (S2) and (*Z*)-1-(4-fluorophenyl)-2-(2-(methylthio)pyrimidin-4-yl)ethanol (S3) *via* 4-methyl-2-(methylthio)pyrimidine (S1)**

Commercially available 2-mercapto-4-methylpyrimidine hydrochloride **1** (10 g, 0.061 mol) was added to a solution of NaOH (4.93 g, 0.123 mol, 2.0 eq.) in H_2_O (85 mL) followed by dropwise addition of methyl iodide (4.22 mL, 0.068 mol, 1.1 eq.). The reaction mixture was stirred at room temperature for 2 hr. It was then quenched by the addition of an aqueous saturated solution of NaHCO_3_. The solution obtained was extracted with DCM (4 x 150 mL). The combined organic extracts were dried over Na_2_SO_4_, filtered and concentrated *in vacuo* to give **S1** as a dark red oil (4.97 g, 96 %).

**^1^H** **NMR** (300 MHz, CDCl_3_) δ 2.44 (3H, s), 2.55 (3H, s), 6.80 (1H, d, *J* = 5.0 Hz), 8.35 (1H, d, *J* = 5.0 Hz). ^1^H NMR data were in accordance with the literature [1].

*^n^*BuLi (75 mL, 0.120 mol, 2.1 eq.) was added dropwise to a solution of dry diisopropylamine (17 mL, 0.120 mol, 2.1 eq.) at – 78 °C under a nitrogen atmosphere. The mixture was then stirred at this temperature for 20 min before being added *via* cannula to a solution of the thiopyrimidine **S1** (8.05 g, 0.057 mol) in anhydrous THF (40 mL). The resulting mixture was stirred at – 78 °C for 1h 30 mins before addition of a solution of methylfluorobenzoate (8.20 mL, 0.063 mol, 1.1 eq.) in anhydrous THF (80 mL). The reaction mixture was then stirred at room temperature overnight and quenched by the addition of an aqueous solution of NH_4_Cl. The mixture was separated and the aqueous layer extracted with EtOAc (3 x 150 mL). The combined organic extracts were then dried over Na_2_SO_4_, filtered and concentrated *in vacuo* to give a mixture of **S2** and **S3** as a dark orange solid. The crude solid was then triturated with hexanes (200 mL) and the solid obtained collected to give a mixture of **S2** and **S3**  (10.10 g, 67 %). **^1^H NMR** (300 MHz, CDCl_3_) δ **S2**: 2.54 (3H, s), 4.37 (2H, s), 7.00 (1H, d, *J* = 5.0 Hz), 7.08 - 7.24 (2H, m), 8.10 (1H, dd, *J* = 9.0, 5.4 Hz), 8.48 (1H, d, *J* = 5.0 Hz); **S3**: 2.64 (3H, s), 5.94 (1H, s), 6.63 (1H, d, *J* = 5.3 Hz), 7.08 - 7.24 (2H, m), 7.78 - 7.91 (2H, m), 8.34 (1H, d, *J* = 5.3 Hz). ^1^H NMR data were in accordance with the literature [1].

**2-Bromo-1-(4-fluorophenyl)-2-(2-(methylthio)pyrimidin-4-yl)ethanone (S4)**

*N*-Bromosuccinimide (11.75 g, 0.066 mol, 1.05 eq.) was added to a suspension of the keto-enol mixture **S2** and **S3** (16.50 g, 0.066 mol) in Et_2_O (230 mL). The reaction mixture was then stirred at room temperature for 3 hr. The mixture was concentrated *in vacuo* to give an orange solid which was isolated, dissolved in DCM (100 mL) and washed with an aqueous solution of sodium thiosulfate (3 x 150 mL). The organic layer was then dried over Na_2_SO_4_, filtered and concentrated *in vacuo* to give a dark oil. Purification by column chromatography (Hexanes/Ethyl acetate: 98/2 – 9/1) afforded the desired product **S4** as a light amber oil (17.41 g, 81 %). **^1^H NMR** (300 MHz, CDCl_3_) δ 2.44 (3H, s), 6.10 (1H, s), 7.10 (2H, t, *J* = 8.6 Hz), 7.32 (1H, d, *J* = 5.1 Hz), 7.94 - 8.10 (2H, m), 8.52 (1H, d, *J* = 5.1 Hz). ^1^H NMR data were in accordance with the literature [1].

**(2-Aminopyridin-4-yl)methanol (S6)**

A solution of lithium aluminium hydride in THF (2 M, 21 mL, 0.04 mol, 1.3 eq.) was added at 0 °C and under a nitrogen atmosphere to a solution of commercially available methyl carboxylate amino pyridine **S5** (5.00 g, 0.03 mol) in anhydrous THF (100 mL). The reaction mixture was then stirred at room temperature for 2 hr. Excess MeOH was added to the flask at 0 °C and the resulting mixture concentrated *in* *vacuo* to give a grey solid. Purification by column chromatography (DCM/MeOH: 9/1) afforded the desired product **S6** as a light yellow solid (3.20 g, 78 %). **^1^H NMR** (300 MHz, CDCl_3_) δ 3.51 (1H, s), 4.47 (2H, br. s.), 4.59 - 4.70 (2H, m), 6.56 (1H, d, *J* = 0.78 Hz), 6.63 (1H, dt, *J* = 5.3, 0.7 Hz), 8.01 (1H, d, *J* = 5.3 Hz). ^1^H NMR data were in accordance with the literature [2].

**(2-(4-Fluorophenyl)-3-(2-(methylthio)pyrimidin-4-yl)imidazo[1,2-a]pyridin-7-yl)methanol (S7)**

A solution of the bromoketone **S4** (687 mg, 22.01 mmol) in EtOH (1 mL) was added to a solution of the aminopyridine **S6** (250 mg, 22.01 mmol) in EtOH (0.75 mL). The reaction mixture was then irradiated at 110 °C in the microwave for 20 min (~ 50 PSI). After cooling, the reaction mixture was concentrated *in vacuo* to give a dark oily solid. Purification by column chromatography (Hexanes/Ethyl Acetate: 95/5 - 0/1) afforded the desired product **S7** as an orange solid (505 mg, 68 %). **^1^H NMR** (400 MHz, CDCl_3_) δ 2.58 (3H, s), 4.74 (2H, s), 6.74 (1H, d, *J* = 5.4 Hz), 6.91 (1H, dd, *J* = 7.2, 1.7 Hz), 7.07 (2H, t, *J* = 8.7 Hz), 7.50 - 7.59 (2H, m), 7.62 (1H, s), 8.23 (1H, d, *J* = 5.4 Hz), 9.48 (1H, d, *J* = 7.2 Hz). ^1^H NMR data are in accordance with the ones reported in the literature [1].

**(2-(4-Fluorophenyl)-3-(2-(methylsulfonyl)pyrimidin-4-yl)imidazo[1,2-a]pyridin-7-yl)methanol (S8)**

*m*CPBA was washed with a buffer solution of Na_2_HPO_4_ (3.5 g in 500 mL, 0.025 mol in H_2_O, pH 8, 50 nM) and NaH_2_PO_4_ (1.38 g in 200 mL of H_2_O, 0.100 mol, pH 5, 50 nM). Freshly washed *m*CPBA was then cooled to 0 °C and stored at this temperature before use.

A suspension of the sulfide **S7** (1.82 g, 4.97 mmol) in anhydrous DCM (60 mL) was cooled to 0 °C and stirred at this temperature for 10 min before addition of *m*CPBA (3.43 g, 19.8 mmol, 4.0 eq.). The reaction mixture was then slowly allowed to warm to room temperature and stirred at this temperature overnight. The bright yellow suspension obtained was concentrated *in vacuo* to give a yellow/orange solid. The crude product was taken up in MeOH (30 mL) and the insoluble solid collected on a glass sinter and washed with MeOH (10 mL). The bright yellow solid collected was then dried under vacuum for 2 hr to yield **S8** as a bright yellow solid (1.30 g, 66 %). **^1^H NMR** (300 MHz, DMSO-*d*_6_) δ 3.47 (3H, s), 4.64 (2H, d, *J* = 5.1 Hz), 5.59 (1H, t, *J* = 5.7 Hz), 7.17 (1H, d, *J* = 7.7 Hz), 7.25 - 7.48 (4H, m), 7.62 - 7.80 (2H, m), 8.81 (1H, d, *J* = 5.5 Hz), 9.49 (1H, d, *J* = 7.7 Hz)). ^1^H NMR data are in accordance with the ones reported in the literature [3].

**(2-(4-Fluorophenyl)-3-(2-(methylsulfonyl)pyrimidin-4-yl)imidazo[1,2-a]pyridin-7-yl)methyl methanesulfonate (S9)**

Dry triethylamine (0.10 mL, 0.733 mmol, 1.5 eq.) was added at room temperature to a solution of the alcohol **S8** (200 mg, 0.502 mmol) in anhydrous THF (5 mL) followed by slow addition of methanesulfonylchloride (0.04 mL, 0.552 mmol, 1.1 eq.) under a nitrogen atmosphere. The reaction mixture was then stirred at room temperature overnight before addition of H_2_O (10 mL). The mixture was extracted with EtOAc (3 x 25 mL) and the combined organic extracts dried over Na_2_SO_4_, filtered and concentrated *in* *vacuo* to give **S9** as a brown/copper solid (230 mg, 96 %). **^1^H NMR** (300 MHz, CDCl_3_) δ 3.12 (3H, s), 3.44 (3H, s), 5.39 (2H, s), 7.15 - 7.26 (3H, m), 7.35 (1H, d, *J* = 5.5 Hz), 7.65 (2H, dd, *J* = 8.4, 5.3 Hz), 7.81 (1H, s), 8.59 (1H, d, *J* = 5.3 Hz), 9.96 (1H, d, *J* = 7.3 Hz). ); **^13^C NMR** (100 MHz, CDCl_3_) 38.3, 39.2, 68.3, 115.0, 115.4, 116.7, 120.7, 128.8, 131.4, 137.2, 157.4, 158.0, 162.7, 165.2, 165.9; **HRMS** (ESI) calculated C_20_H_18_N_4_O_5_FS_2_ 477.0703 found 477.0697 (- 1.1ppm).

**1-(2-(4-Fluorophenyl)-3-(2-(methylsulfonyl)pyrimidin-4-yl)imidazo[1,2-a]pyridin-7-yl)-*N,N*-dimethylmethanamine (2)**

Dry diisopropylethylamine (0.31 mL, 1.69 mmol, 1.3 eq.) was added under a nitrogen atmosphere to a solution of the mesylate **S9** (615 mg, 1.29 mmol) in anhydrous CH_3_CN (5 mL) followed by a solution of dimethylamine in THF (2 M, 0.68 mL, 1.42 mmol, 1.1 eq.). The reaction mixture was then stirred at room temperature overnight. DCM (3 mL) and H_2_O (3 mL) were then added to the flask and the resulting mixture left to stir vigorously for 30 min. The two layers were then separated and the aqueous layer was extracted with DCM (3 x 25 mL). The combined organic extracts were then dried over Na_2_SO_4_, filtered and concentrated *in vacuo* to give **2** as a copper-coloured solid (460 mg, 84 %). **^1^H NMR** (300 MHz, CDCl_3_) δ 2.24 (6H, s), 3.33 (3H, s), 7.09 - 7.17 (4H, m), 7.46 - 7.64 (3H, m), 8.44 (1H, d, *J =* 5.63 Hz), 9.75 (1H, d, *J* = 7.17 Hz). ^1^H NMR data are in accordance with the ones reported in the literature [1].

**Compound 2: 4-(7-((Dimethylamino)methyl)-2-(4-fluorophenyl)imidazo[1,2-a]pyridin-3-yl)pyrimidin-2-amine**

A suspension of **2** (50 mg, 0.117 mmol) in a solution of ammonia in 2-propanol (2M, 3.35 mL, 6.70 mmol, 57 eq.) was irradiated for 4 hr at 100 °C in a microwave (PSI ~ 100). The reaction mixture was then concentrated *in vacuo* to give a light brown solid. Purification by column chromatography (DCM/MeOH/Et_3_N: 99/0/1 – 97/2/1) followed by trituration with ethyl acetate and then hexanes afforded the desired **Compound 2** as an off white solid (10 mg, 24 %). **^1^H NMR** (300 MHz, CDCl_3_) δ 2.23 (6H, s), 3.45 (2H, s), 5.06 (2H, br. s.), 6.45 (1H, d, *J* = 5.2 Hz), 6.94 (1H, d, *J* = 7.2 Hz), 7.05 (2H, t, *J* = 8.6 Hz), 7.48 (1H, s), 7.57 (2H, dd, *J* = 8.6, 5.2 Hz), 8.05 (1H, d, *J* = 5.2 Hz), 9.34 (1H, d, *J* = 7.2 Hz). ^1^H NMR data are in accordance with the ones reported in the literature [1].

1. **Synthesis of MTX-Cmpd2.1**
   1. **Synthesis of Compound 2 – linker S11**

**Scheme S2**: Synthesis of Compound 2 – linker **S11**

***tert*-Butyl 17-(4-(7-((dimethylamino)methyl)-2-(4-fluorophenyl)imidazo[1,2-a]pyridin-3-yl)pyrimidin-2-ylamino)-3,6,9,12,15-pentaoxaheptadecylcarbamate (S11)**

A solution of **2** (150 mg, 0.35 mmol) and linker *tert*-butyl (17-amino-3,6,9,12,15-pentaoxaheptadecyl)carbamate [[39](#_ENREF_39)] **S10** (330 mg, 0.87 mmol, 2.5 eq.) in acetonitrile (2 mL) was stirred for 4 hr at 100 °C in the microwave. After cooling, the reaction mixture was concentrated *in vacuo*. Purification by flash chromatography (DCM/MeOH: 100/0 – 94/6) afforded the expected compound **S11** as a yellow oil (190 mg, 74 %). **IR νmax** (cm^-1^): 814, 844, 1099, 1249, 1505, 1574, 1714, 2865, 3353; **^1^H NMR** (400 MHz, CDCl_3_) δ 1.42 (9H, s); 2.33 (6H, s), 3.29-3.32 (2H, m), 3.61-3.87 (24H, m), 5.11 (1H, br. s), 5.73 (1H, br. s), 6.41 (1H, d, *J* = 5.2 Hz), 7.04 (1H, d, *J* = 7.2 Hz), 7.11-7.14 (2H, m), 7.54 (1H, s), 7.63-7.67 (2H, m), 8.09 (1H, d, *J* = 5.2 Hz), 9.44 (1H, d, *J* = 7.2 Hz); **^13^C NMR** (100 MHz, CDCl_3_) δ 28.4, 29.7, 40.4, 41.4, 45.2, 63.3, 70.6-70.0, 109.8, 114.3, 115.6, 116.5, 118.5, 127.0, 130.8, 131.2, 146.4, 147.8, 156.0, 162.2; **HRMS** (ESI) calculated C_37_H_52_N_7_O_7_FNa 748.3810 found 748.3818 (1.0 ppm).

- 1. **Synthesis of *^t^*Butyl methotrexate 5**

**Scheme S3**: Synthetic route to *^t^*butyl methotrexate **5**

**4-(((2,4-Diaminopteridin-6-yl)methyl)(methyl)amino)benzoic acid (S12)**

Pteridine alcohol hydrochloride (**4**) (559 mg, 2.26 mmol) was dissolved in anhydrous DMA (7 mL) and triphenylphosphine dibromide (2.87 g, 6.80 mmol, 3.0 eq.) was added. The reaction mixture was then stirred at room temperature for 24 hr. 4-(Methylamino)benzoic acid (429 mg, 2.84 mmol, 1.3 eq.) was then added followed by dry diisopropylethylamine (1.18 mL, 6.80 mmol, 3.0 eq.). The reaction was stirred at room temperature for a further 72 hr, poured onto an aqueous solution of NaOH (0.33 M, 50 mL) and the resulting precipitate isolated by filtration and washed with H_2_O (5 mL). The mother liquor was then acidified to pH 5.5 by the addition of an aqueous solution of AcOH (10 %). The resulting light brown solid was collected by filtration and dried in a vacuum oven for 4 hr (50 °C) to give **S12** as an orange solid (676.3 mg, 92 %). **^1^H NMR** (300 MHz, DMSO-*d*_6_) δ 3.24 (3H, s), 4.82 (2H, s), 6.83 (2H, d, *J* = 8.96 Hz), 7.09 (2H, br. s.), 7.73 (2H, d, *J* = 8.96 Hz), 8.21 (2H, br. s.), 8.64 (1H, s), 12.12 (1H, br. s.). ^1^H NMR data were in accordance with the literature [5].

**(*R*)-5-(*tert*-Butoxy)-4-(4-(((2,4-diaminopteridin-6-yl)methyl)(methyl)amino)benzamido)-5-oxopentanoic acid (5)**

Pteridic acid **S12** (645 mg, 1.99 mmol) was stirred in anhydrous DMF (10 mL) in the presence of triethylamine (554 mL, 3.98 mmol, 2.0 eq.) for 15 min until complete dissolution of the acid had occurred. TPTU (591 mg, 1.99 mmol) was then added to the flask and the solution left to stir at room temperature overnight. In a separate flask, L-glutamic acid *^t^*butyl ester (451 mg, 2.22 mmol, 1.1 eq.) and triethylamine (554 mL, 3.98 mmol, 2.0 eq.) were suspended in anhydrous DMF (5 mL). The activated acid was then added *via* cannula to the ester and the reaction mixture left to stir at room temperature overnight. The reaction mixture was concentrated *in vacuo* and the residue triturated with ethyl acetate. The resulting solid was collected via filtration, washed with ethyl acetate (1 x 20 mL) and CHCl_3_ (3 x 20 mL) and dried at 40 °C under vacuum to give the desired compound **5** as an orange solid (826 mg, 81 %). ^1^H NMR (300 MHz, DMSO-*d*_6_) δ 1.38 (9H, s), 1.99-2.05 (2H, m), 2.31 (2H, t, *J* = 7.3 Hz), 3.22 (3H, s), 4.24-4.26 (1H, m), 4.81 (2H, s), 6.81 (2H, d, *J* = 8.8 Hz), 7.72 (2H, d, *J* = 8.8 Hz), 8.19 (1H, d, *J* = 7.5 Hz), 8.36 - 8.54 (1H, m), 8.63 (1 H, s). ^1^H NMR data were in accordance with the literature [5].

1. **Synthesis of MTX-Cmpd2.1**

**Scheme S4:** Synthetic route to **MTX-Cmpd2.1**

***tert*-Butyl ester MTX-Cmpd2.1 S13**

A solution of **S11** (190 mg, 0.260 mmol) in TFA (500 μL) was stirred overnight at room temperature. The reaction mixture was then evaporated to dryness and co-evaporated with cyclohexane to afford the free amine **3** as an orange oil.

A mixture of carboxylic acid **5** (133 mg, 0.260 mmol), TPTU (78 mg, 0.260 mmol) and dry DIPEA (180 μL, 1.05 mmol, 4.0 eq.) in dry DMF (8 mL) was stirred for 30 min at room temperature. A solution of **3** (123 mg, 0.260 mmol) in dry DMF (8 mL) was then added. The resulting reaction mixture was stirred for 36 hr at room temperature. After evaporation of the solvent, the residue was directly purified by flash chromatography (DCM/MeOH/Et_3_N: 98/0/2 to 98/6/2) to afford the desired product **S13** as a yellow oil (180 mg, 62 %). **IR νmax** (NaCl, cm^-1^): 1098, 1155, 1365, 1459, 1509, 1571, 1608, 1638, 2925; **^1^H NMR** (400 MHz, CDCl_3_) δ 1.43 (9H, s), 2.26 (6H, s), 3.44 (3H, s), 3.72-3.47 (30H, m), 4.56-4.59 (1H, m), 4.69 (2H, s), 6.39 (1H, d, *J* = 5.2 Hz), 6.39 (1H, d, *J* = 5.2 Hz), 6.68 (2H, d, *J* = 8.8 Hz), 6.80 (1H, br. s.), 6.96 (1H, d, *J* = 7.2 Hz), 7.09 (2H, t, *J* = 8.8 Hz), 7.14 (1H, d, *J* = 7.6 Hz), 7.62 (2H, m), 7.53 (1H, s), 7.68 (2H, d, *J* = 8.8 Hz), 8.06 (1H, d, *J* = 5.2 Hz), 8.55 (1H, s), 9.38 (1H, d, *J* = 7.2 Hz); **^13^C NMR** (100 MHz, CDCl_3_) 28.1, 39.3, 39.5, 41.4, 45.9, 53.1, 55.9, 63.5, 70.5-69.6, 82.1, 111.5, 114.3, 115.6, 116.3, 121.8, 122.0, 126.8, 128.9, 131.2, 139.9, 146.5, 146.7, 149.5, 151.3, 155.3, 157.5, 157.8, 158.0, 162.2, 162.6, 163.1, 167.0, 171.6, 172.7; **LRMS** (ESI): calculated [M+H]^+^ = 1117.56, found [M+H]^+^ = 1118.11.

**22-(4-(((2,4-Diaminopteridin-6-yl)methyl)(methyl)amino)benzamido)-1-(4-(7- ((dimethylamino)methyl)-2-(4-fluorophenyl)imidazo[1,2-a]pyridin-3-yl)pyrimidin-2-ylamino)-19-oxo-3,6,9,12,15-pentaoxa-18-azatricosan-23-oic acid, MTX-Cmpd2.1**

**

Thioanisole (88 μL) and water (88 μL) were added to a solution of **S13** (90 mg, 0.08 mmol) in TFA (1.6 mL). The reaction mixture was stirred overnight at room temperature and then evaporated to dryness. Purification by reverse phase chromatography (H_2_O/MeOH: 100/0 – 0/100) afforded the expected **MTX-Cmpd2.1** as an oily yellow solid (50 mg, 59 %). **IR νmax** (NaCl, cm^-1^): 704, 738, 843, 950, 1096, 1266, 1350, 1450, 1501, 1572, 1648, 2925, 3055, 3339; **^1^H NMR** (400 MHz, MeOD) δ 2.35 (6H, s), 3.20 (3H, s), 3.23-3.70 (30H, m), 4.43-4.45 (1H, m), 4.59 (2H, s), 6.33 (1H, d, *J* = 4.4 Hz), 6.82 (2H, d, *J* = 6.4 Hz), 7.11 (1H, d, *J* = 5.6 Hz), 7.21 (2H, t, *J* = 6.0 Hz), 7.58 (1H, s), 7.60-7.63 (2H, m), 7.72 (2H, d, *J* = 6.0 Hz), 8.06 (1H, d, *J* = 3.6 Hz), 8.55 (1H, s), 9.61 (1H, br. s); **LRMS** (ESI): calculated [M+H]^+^ = 1061.56, found [M+H]^+^ = 1062.06, [M+Na]^+^ = 1083.99, [M-H]^-^ = 1060.08.

1. **Synthesis of MTX-Cmpd1.1**

**Scheme S5:** Synthetic route to **MTX-Cmpd1.1**

***tert*-Butyl 4-(4-(4-fluorophenyl)-4-oxo-3-(pyridin-4-yl)butanoyl)piperidine-1-carboxylate (S15) *via* 1-(4-Fluorophenyl)-2-(pyridine-4-yl)ethanone (S18)** [6]

To a mixture of commercially available 4-methylpyridine (5.0 g, 53.7 mmol) and ethyl 4-fluorobenzoate (9.0 g, 53.7 mmol) in THF (40 mL) was added dropwise LHMDS (53.5 mL, 1.0 M solution in THF) at 0 °C under a nitrogen atmosphere. After stirring for 1 hr, the reaction mixture was allowed to warm to room temperature, triturated with hexanes (140 mL) and filtered. The solid was dissolved in 3N HCl (50 mL). The solution obtained was neutralised with a saturated aqueous solution of NaHCO_3_ and extracted with ethyl acetate (3 x 75 mL). The combined organic extracts were washed with water (150 mL) and brine (100 mL), dried over MgSO_4_ and concentrated *in* *vacuo* to afford the desired product **S18** as a light yellow solid (10 g, 91 %). **^1^H NMR** (300 MHz, CDCl_3_): δ 4.25 (2H, s), 7.10-7.21 (4H, m), 7.80-7.90 (2H, m), 8.52-8.55 (2H, m). ^1^H NMR data were in accordance with the literature [7].

Sodium hydride (95%, 0.11 g, 4.88 mmol, 1.05 eq.) was added to anhydrous DMSO (5 mL) at 10 °C under a nitrogen atmosphere. The mixture was stirred at this temperature for 15 min before addition of a solution of **S18** (1.00 g, 4.65 mmol) in DMSO (5 mL). The reaction mixture was then stirred for 15 min at this temperature before addition of a solution of *tert*-butyl 4-(2-bromoacetyl)piperidine-1-carboxylate [8] (1.49 g, 4.88 mmol, 1.05 eq.) in DMSO (10 mL) over a period of 15 min at 10 °C. The reaction mixture was then stirred at this temperature for 2 hr before being allowed to warm up to room temperature overnight. The reaction mixture was diluted with a mixture of water/brine (50/50) and extracted with ethyl acetate (3 x 50 mL). The combined organic extracts were washed with brine (100 mL), dried over MgSO_4_ and concentrated *in vacuo* to afford the desired crude product **S15**. Purification by column chromatography (EtOAc/Hexanes: 9/1) afforded **S15** as an orange oil (0.90 g, 47 %). **IR νmax** (neat, cm^-1^) 3401, 2972, 2927, 2857, 1679, 1594, 1557, 1508, 1443, 1421, 1363, 1276, 1237, 1127, 1068, 1008, 939, 844, 817, 770, 603, 576, 536; **^1^H NMR** (300 MHz, CDCl_3_): δ 1.45 (9H, s), 1.58-1.62 (2H, m), 1.74-1.84 (1H, m), 1.84-1.94 (1H, m), 2.58-2.61 (1H, m), 2.78-2.90 (3H, m), 3.66-3.71 (1H, m), 5.05-5.10 (1H, m), 7.05-7.15 (2H, m), 7.25-7.15 (2H, m), 7.97-8.01 (2H, m), 8.58-8.61 (2H, m); **HRMS** (ES) calculated C_25_H_29_N_2_O_4_FNa 463.1998 found 463.1997 (- 0.1 ppm).

**Compound 1-linker S17 *via tert*-Butyl 4-(5-(4-fluorophenyl)-4-(pyridine-4-yl)-1*H*-pyrrol-2-yl)piperidine-1-carboxylate S19**

To a solution of **S15** (1.0 g, 2.27 mmol) in acetic acid (10 mL) was added solid ammonium acetate (10 g) at room temperature. The reaction mixture was heated 115 °C for 12 hr. The reaction mixture was diluted with water/brine (100/50 mL) and extracted with ethyl acetate (3 x 50 mL). The combined organic layers were washed with brine (2 x 50 mL), dried over MgSO_4_ and concentrated *in vacuo* to afford desired crude product **S19**. Purification by column chromatography (EtOAc/Hexanes: 9/1) afforded **S19** as a yellow solid (0.5 g, 55 %). **IR νmax** (KBr, cm^-1^) 2977, 2748, 1684, 1594, 1525, 1510, 1473, 1448, 1418, 1271, 1229, 1165, 1110, 1030, 996, 834, 772, 683, 608, 573, 536; **^1^H NMR** (300 MHz, CDCl_3_): δ 1.45 (9H, s), 1.58-1.62 (2H, m), 1.84-1.94 (2H, m), 2.68-2.82 (3H, m), 4.00-4.13 (2H, m), 6.15 (1H, s), 6.96-7.01 (2H, m), 7.25-7.35 (4H, m), 8.15-8.25 (2H, m), 11.15 (1H, br. s); **HRMS** (ES) calculated C_25_H_29_N_3_O_2_F 423.2244 found 423.2241 (- 0.3 ppm).

To a solution of **S19** (1.0 g, 2.37 mmol) in DCM (20 mL) was added trifluoroacetic acid (2.0 mL) at 0 °C under a nitrogen atmosphere. The reaction mixture was stirred at 0 °C for 1 hr and at room temperature for 1 hr. The reaction mixture was diluted with water (20 mL) and the organic layer separated. The aqueous layer was extracted with ethyl acetate (2 x 30 mL). The combined organic extracts were washed with a saturated solution of sodium bicarbonate (20 mL) and brine (20 mL), dried over gSO_4_ and concentrated *in vacuo*. The crude product was purified by trituration in DCM (10 mL) to afford the free amine as a light yellow solid.

The free amine (0.50 g, 1.55 mmol) was taken up in anhydrous DMF (4 mL) and a solution of PEG6 linker **S16** [[39](#_ENREF_39)] (0.85 g, 1.86 mmol, 1.2 eq.) in anhydrous DMF (4 mL) was added to the flask followed by DIPEA (8.0 mL, 46.5 mmol, 30 eq.). The reaction mixture was stirred at room temperature overnight under a nitrogen atmosphere. The solvent was then removed *in vacuo* to yield a brown oil. Purification by column chromatography (DCM/ MeOH: 8/2) afforded **S17** as a bright yellow oil (0.22 g, 21 %). **IR νmax** (neat, cm^-1^) 3453, 3147, 2926, 2862, 2251, 1791, 1705, 1636, 1599, 1513, 1454, 1388, 1365, 1348, 1282, 1223, 1174, 1100, 1039, 906, 839, 729, 648, 549; **^1^H NMR** (300 MHz, CDCl_3_): δ 1.35 (9H, s), 1.95-2.05 (2H, m), 2.15-2.20 (2H, m), 2.56-2.60 (1H, m), 2.65-2.75 (2H, m), 2.85-2.95 (2H, m), 3.15-3.25 (2H, m), 3.33-3.40 (2H, m), 3.41-3.45 (2H, m), 3.45-3.65 (16H, m), 3.78-3.83 (2H, m), 4.98-5.08 (1H, m), 6.1 (1H, s), 6.97-7.02 (2H, m), 7.16-7.20 (2H, m), 7.25-7.35 (2H, m), 8.37-8.42 (2H, m), 9.35 (1H, br. s).

***tert*-Butyl MTX-Cmpd1.1**

**S17** (40 mg, 0.584 mmol) was dissolved in DCM (15 mL) and TFA (0.5 mL) added to the flask. The reaction mixture was stirred for 0.5 hr before removal of the solvent *in vacuo* to yield the free amine as a dark brown oil.

TPTU (95 mg, 0.323 mmol, 1.1 eq.) was added to a solution of the free amine (200 mg, 0.293 mmol) in anhydrous DMF (4 mL) followed by DIPEA (1.0 mL, 5.87 mmol, 15 eq.) and a solution of ^t^butyl MTX **5** (169 mg, 0.323 mmol, 1.1 eq.) in anhydrous DMF (4 mL). The reaction mixture was stirred at room temperature for 2 days before being concentrated *in vacuo* to yield a brown solid. Purification by column chromatography (DCM/MeOH/TEA 9/1/1) afforded ***^t^*butyl MTX-Cmpd1.1** as a bright yellow oil (53 mg, 17 %). **IR νmax** (neat, cm^-1^) 3431, 3077, 2932, 2842, 2778, 1701, 1654, 1594, 1527, 1508, 1475, 1448, 1381, 1279, 1234, 1214, 1152, 1120, 1088, 1068, 998, 956, 837, 824, 812, 762, 685, 615, 581, 514; **^1^H NMR** (300 MHz, DMSO-*d_6_*): δ 1.45 (9H, s), 1.68-1.72 (2H, m),1.85-1.95 (2H, m), 1.95-2.05 (2H, m), 2.15-2.25 (1H, m,), 2.68-2.78 (1H, m), 2.88-2.98 (2H, m), 3.15-3.20 (2H, m), 3.22 (3H, s), 3.45-3.55 (26H, m), 4.28-4.38 (1H, m), 4.84 (2H, s), 6.11 (1H, s), 6.63 (2H, br. s), 6.78-6.88 (2H, m), 7.12-7.22 (2H, m), 7.22-7.32 (2H, m), 7.32-7.42 (2H, m), 7.65-7.75 (2H, m), 8.38-8.42 (2H, m), 8.55 (1H, s), 11.11 (1H, br. s).

**MTX-Cmpd1.1**

***^t^*Butyl MTX-Cmpd1.1** (180 mg, 0.167 mmol) was dissolved in DCM (10 mL), TFA (0.5 mL) was then added to the flask. The resulting solution was stirred at room temperature for 7 hr before being concentrated *in vacuo* to yield crude **MTX-Cmpd1.1**. Purification by column chromatography (DCM/MeOH/TEA: 9/1/1) afforded **MTX-Cmpd1.1** as an oil (68 mg, 40%). **IR νmax** (neat, cm^-1^) 3431, 3027, 2852, 2753, 2523, 2354, 1699, 1672, 1599, 1527, 1512, 1423, 1202, 1175, 1135, 1001, 956, 834, 717, 688; **^1^H NMR** (300 MHz, DMSO-*d_6_*): δ 1.61-1.71 (2H, m), 1.84-1.94 (2H, m), 1.95-2.05 (2H, m), 2.14-2.24 (1H, m), 2.76-2.81 (1H, m), 2.88-2.98 (2H, m), 3.11-3.16 (2H, m), 3.22 (3H, s), 3.45-3.55 (26H, m), 4.22-4.32 (1H, m), 4.81 (2H, s), 6.10 (1H, s), 6.61 (2H, br. s), 6.78-6.88 (2H, m), 7.12-7.22 (2H, m), 7.22-7.32 (2H, m), 7.32-7.42 (2H, m), 7.65-7.75 (2H, m), 8.34-8.40 (2H, m), 8.55 (1H, s), 11.11 (1H, br. s).

**References**

1. Scribner A, Dennis R, Hong J, Lee S, McIntyre D, et al. (2007) Synthesis and biological activity of imidazopyridine anticoccidial agents: part I. Eur J Med Chem 42: 1334-1357.

# 2. Selwood D L, Brummell D G, Budworth J, Burtin G E, Campbell R O, et al. (2001) Synthesis and biological evaluation of novel pyrazoles and indazoles as activators of the nitric oxide receptor, soluble guanylate cyclase. J Med Chem 44: 78-93.

3. Scribner A, Dennis R, Lee S, Ouvry G, Perrey D, et al. (2008) Synthesis and biological activity of imidazopyridine anticoccidial agents: part II. Eur J Med Chem 43: 1123-1151.

4. Walton J G A, Patterson S, Liu G, Haraldsen J D, Hollick J J, et al. (2009) Synthesis and biological evaluation of functionalised tetrahydro-β-carboline analogues as inhibitors of Toxoplasma gondii invasion*.* Org Biomol Chem 7: 3049-3060.

5. Francis C L, Yang Q, Kart N K, Widmer F, Manthey M K, He-Williams H M (2002) [Total synthesis of methotrexate-γ-TRIS-fatty acid conjugates](http://www.publish.csiro.au/paper/CH02125.htm). Australian J Chem 55: 635-645.

6. Bagley M C, Davis T, Rokicki M J, Widdowson C S, Kipling D (2010) Synthesis of the highly selective p38 MAPK inhibitor UR-13756 for possible therapeutic use in Werner syndrome. Future Med Chem 2: 193-201.

7. Peifer C, Kinkel K, Abadleh M, Schollmeyer D, Laufer S (2007) From five- to six-membered rings: 3,4-diarylquinolinone as lead for novel p38 MAP kinase inhibitors. J Med Chem 50: 1213-1221.

8. Butora G, Alexander P (2004) gamma-Aminoamide modulators of chemokine receptor activity. WIPO International Patent Application WO2004/041279 A1.
