# Supplementary material for: Yeast Three-Hybrid Screen Identifies TgBRADIN/GRA24 as a Negative Regulator of Toxoplasma gondii Bradyzoite Differentiation
Source: PLoS One. 2015 Mar 19;10(3):e0120331. doi: 10.1371/journal.pone.0120331 (PMC4366382; doi:10.1371/journal.pone.0120331)
Supplement: S1 Table — (DOC) [file pone.0120331.s008.doc]

| Primer | Name | Sequence (5’🡪3’) |
| --- | --- | --- |
| 1 | Tg*CK1a*@start *Mfe*I Fw | ATTACAATTGATGGAGGTCAGGGTCGGAGGC |
| 2 | Tg*CK1a*@stop *Mfe*I Rev | CGCAATTGTTACTAcatcggcaactcgTTCTG |
| 3 | Tg*PKG*@ int start *Mfe*I Fw | TACAATTGATGAACTCTCCCAAGAC |
| 4 | Tg*PKG*@stop 3 frame *Mfe*I Rev | ATCGCAATTGTCACTTACCTATTATCAGAAATCCTTGTCC |
| 5 | Tg*CDPK1* IVC pJG@849bp Fw | TATGATGTGCCAGATTATGCCTCTCCCGAAATGGGGCAGCAGGAAAGC |
| 6 | Tg*CDPK1* IVC pJG @849bp Rev | ACAAGCCGACAACCTTGATTGGAGACTTGATTAgtttccgcagagcTTCA |
| 7 | Tg*BRADIN*@792bp Fw | CAGGCACAATAATCCCCCTCGTG |
| 8 | Tg*BRADIN*ORF1@stop Rev | TTATCAGTGCGAGAGTGCTCCCCG |
| 9 | Tg*CDPK1*@start *Eco*RI Fw | ATTAGAATTCATGGGGCAGCAGGAAAGC |
| 10 | Tg*CDPK1*@1234bp Rev | GAGTGCCTTGGAACTGTCGG |
| 11 | Tg*BRADIN*@805bp Fw | ATAATGCCCCTCGTGCTATTGCCCG |
| 12 | Tg*BRADIN*@1624bp *Avr*II Rev | CAGTGCCTAGGATTACCCTTAGTGGGTGGTTTAAC |
| 13 | Tg*BRADIN*@-516bp *Kpn*I Fw | CAGTGGTACCTCTTCAGACTCTTGAGC |
| 14 | Tg*BRADIN*@-30bp *Hind*III Rev | AGTCAAGCTTTGGGTGCGACGATTC |
| 15 | Tg*BRADIN*@+144bp *Bam*HI Fw | CATCGGATCCCACATTGTTTGCTACG |
| 16 | Tg*BRADIN*@+809bp *Xba*I Rev | CTGAATCTAGAACGACTTCCACACAAAGAGG |
| 17 | Tg*BRADIN*@1112bp-exon4 Fw | CGCAGTGTTCTGGAAGGACTCACG |
| 18 | Tg*BRADIN*@stopORF2 Rev | CGTGTTAATTACCCTTAGTG |
| 19 | Tg*BRADIN*@-556bp P5 Fw | TAGCCAAATAGAGCAACACCCTG |
| 20 | Tg*BRADIN*@+409bp KO.P2 Rev | TCAACAGAAAGTGCCACCAC |
| 21 | GRA1.5’UTR Rev | CCGCTTACGGCGTCTCATACC |
| 22 | F10 | TGCGTTTGCGGATGCGGAGTTC |
| 23 | F9 | GGTCACCGAAATCACCAATGC |
| 24 | Ble ORF Probe For | CCGACCAAGCGACGCCCAACCTGCC |
| 25 | Ble ORF Probe Rev | CGTTCTGTATCAGGCGCAGGAGCG |

**Table S1: Primers used in this study**
