# Supplementary material for: Yeast Three-Hybrid Screen Identifies TgBRADIN/GRA24 as a Negative Regulator of Toxoplasma gondii Bradyzoite Differentiation
Source: PLoS One. 2015 Mar 19;10(3):e0120331. doi: 10.1371/journal.pone.0120331 (PMC4366382; doi:10.1371/journal.pone.0120331)
Supplement: S2 Table — (DOC) [file pone.0120331.s009.doc]

**Table S2: *Toxoplasma gondii* strains used in this study**

| Strain | Genotype | Source/Reference |
| --- | --- | --- |
| Wild-type | RH Δ*ku80* Δ*hxgprt* | [38,39] |
| Δ*uprt* | RH Δ*ku80* Δ*hxgprt* Δ*uprt* | This study |
| Δ*bradin* | RH Δ*ku80* Δ*hxgprt* Δ*bradin* | This study |
| Δ*bradin* Δ*uprt* | RH Δ*ku80* Δ*hxgprt* Δ*bradin* Δ*uprt* | This study |
